# Silicon Carbide (SiC) and Silicon/Carbon (Si/C) Composites for High-Performance Rechargeable Metal-Ion Batteries

**DOI:** 10.3390/ijms26167757

**Published:** 2025-08-11

**Authors:** Sara Adnan Mahmood, Nadhratun Naiim Mobarak, Arofat Khudayberdieva, Malika Doghmane, Sabah Chettibi, Kamel Eid

**Affiliations:** 1 Department of Chemical Sciences, Faculty of Science and Technology, University Kebangsaan Malaysia, Bangi 43600, Selangor, Malaysia; 2 Gas Processing Center (GPC), College of Engineering, Qatar University, Doha 2713, Qatar; 3 Physics and Energy Department, Tashkent Chemical-Technological Institute, Tashkent 100011, Uzbekistan; fek@tkti.uz; 4 Laboratoire de Physique des Matériaux, Université 8 Mai Guelma 1945, Guelma 24000, Algeria; malika_doghmane@yahoo.fr (M.D.); chettibisabah05@yahoo.fr (S.C.)

**Keywords:** SiC, batteries, Li-ion, Zn-ion, Na-ion, composite

## Abstract

Silicon carbide (SiC) and silicon nanoparticle-decorated carbon (Si/C) materials are electrodes that can potentially be used in various rechargeable batteries, owing to their inimitable merits, including non-flammability, stability, eco-friendly nature, low cost, outstanding theoretical capacity, and earth abundance. However, SiC has inferior electrical conductivity, volume expansion, a low Li^+^ diffusion rate during charge–discharge, and inevitable repeated formation of a solid–electrolyte interface layer, which hinders its commercial utilization. To address these issues, extensive research has focused on optimizing preparation methods, engineering morphology, doping, and creating composites with other additives (such as carbon materials, metal oxides, nitrides, chalcogenides, polymers, and alloys). Owing to the upsurge in this research arena, providing timely updates on the use of SiC and Si/C for batteries is of great importance. This review summarizes the controlled design of SiC-based and Si/C composites using various methods for rechargeable metal-ion batteries like lithium-ion (LIBs), sodium-ion (SIBs), zinc-air (ZnBs), and potassium-ion batteries (PIBs). The experimental and predicted theoretical performance of SiC composites that incorporate various carbon materials, nanocrystals, and non-metal dopants are summarized. In addition, a brief synopsis of the current challenges and prospects is provided to highlight potential research directions for SiC composites in batteries.

## 1. Introduction

The ever-increasing energy crisis has been caused by the excessive utilization of fossil fuel, leading to its depletion and subsequent numerous environmental detriments such as global warming and climate change [1,2]. In response, the necessity for green electrochemical energy storage and conversion technologies has escalated significantly over the last few decades [3]. The developed fuel cells (i.e., methanol, ethanol, and oxygen-reduction) [4,5], along with water-splitting [6,7] and energy storage devices, emerged as potential solutions. Energy storage devices, such as batteries and supercapacitors, allow for the efficient harnessing of intermittent energy sources, as well as upsurges in energy conversion performance [8]. Various metal-ion batteries including lithium-ion (LIBs), sodium-ion (NIBs), zinc-air (ZnBs), and potassium-ion batteries (PIBs) were developed [9,10,11,12,13,14]. However, LIBs are the most widely used in practical applications, including, but not limited to, aerospace, electric cars [15], medical facilities [16], and military equipment [17]. This is due to the obvious pros of LIBs, such as outstanding energy density, impressive rate performance, long-term cycle life, and inferior self-discharge rate [18,19].

The battery performance (i.e., specific capacity, life cycle, and energy density) is shaped by the type of anode, cathode, and electrolyte. Various anodes were developed for batteries like metal oxide, alloys, metal oxalate, chalcogenide, and metal carbide, which varied in their performance and price [20,21]. Owing to their earth abundance, eco-friendly nature, low cost, high theoretical capacity (~4200 mAh/g), and inferior delithiation potential (~0.4 V vs. Li/Li^+^), Si-based anode materials have attracted great interest for use in the next generation of inexpensive and durable batteries [22,23,24]. Also, Si can accommodate up to fifteen Li ions per four Si atoms, which is significantly superior to graphene anodes (one Li/six C atoms) [25,26]. However, the inevitable volume expansion of Si can reach nearly 400% during a charge–discharge cycle, causing subsequent cracking and loss of activity [27]. In addition to the direct contact of Si and the electrolyte, undesirable side reactions cannot be avoided; meanwhile, the repeated failure and creation of a solid–electrolyte interphase during charge/discharge cycles reduces the active Li-content [28]. Moreover, the inferior electrical/ionic conductivities of Si slow down the charge and discharge rates and boost the resistance at the electrode’s surface [29,30]. These limitations are the main barriers precluding the practical applications of Si-based anodes for use in batteries. In order to combat these barriers, various solutions and ongoing research efforts have been contributed, culminating in the discovery of a controlled structure [31,32], forming a composite with carbon materials [33], electrolyte optimization [34,35,36], binder improvements [37], and interface engineering [38,39].

Coupling silicon (Si) with carbon in the form of composites, core–shell structures, coatings, and carbides is one of the most promising approaches to prevent Si volume expansion by acting as a buffer, enhancing electrical conductivity, and reducing contact with the electrolyte [40,41]. Silicon carbide (SiC) is a semiconductor material consisting of silicon (Si) atoms that are chemically bonded to carbon (C) atoms through strong covalent bonds, maintaining a fixed ratio of 1:1 within a crystalline or polycrystalline lattice [42]. In contrast, the Si/C composite material is made up of silicon nanoparticles confined within a carbon phase with variable ratios, achieved through physical combinations or engineered structures such as core–shell configurations, matrices, or coatings [43]. The crystalline structure of SiC can exist in various polytypes, including 4H, 6H, and 3C, while the Si/C composite features amorphous carbon alongside crystalline silicon, with a variable ratio between the two components. Silicon carbide (SiC) and Si/C offer several advantages, including high thermal conductivity, high permittivity, a quicker electron saturation drift velocity, a higher melting point, and greater Mohs hardness. Additionally, SiC demonstrates promising electrical, chemical, and electrochemical properties, and is highly compatible with current microfabrication techniques. SiC has a bandgap energy that is three times greater than that of silicon, resulting in reduced intrinsic carrier transformation, increased breakdown field strength, and a tenfold higher electric field compared to silicon, making SiC a favorable anode material for batteries [44,45,46]. Furthermore, SiC-based devices can operate in various harsh environments due to their outstanding mechanical properties and chemical durability. Notably, the layered lattice structure of SiC can enhance the diffusion of small ions while providing abundant interstitial sites for accommodating metal ions (e.g., lithium and sodium). This structure can improve electrical conductivity and capacity, enhance structural flexibility, prolong battery life cycles, and prevent Si volume expansion in various metal-ion batteries, particularly lithium-ion batteries (LIBs) [41]. The performance of SiC and Si/C anodes in metal-ion batteries can be enhanced significantly via doping SiC with various dopants (i.e., metal, non-metal, and hybrid) [47] and coating with carbon materials (i.e., carbon, graphene, carbon nanotubes) [48,49]. The latter is considered the most promising because it enhances electrical conductivity, improves the interaction with the electrolyte, and stabilizes SiC against volume changes through the use of carbon materials [50]. Thus, various methods have been developed for the formation of SiC composites, such as ball milling, pyrolysis, spray drying, and chemical vapor deposition; these also promote battery performance. There are various reviews underlining the advances in the synthesis of Si-based anodes for batteries; however, as far as we have found, the effects of SiC composites—with and without additives or dopants—on various batteries have not been sufficiently explored. Therefore, it is crucial to provide timely updates on this research area [28,51,52,53,54].

**Figure 1:**
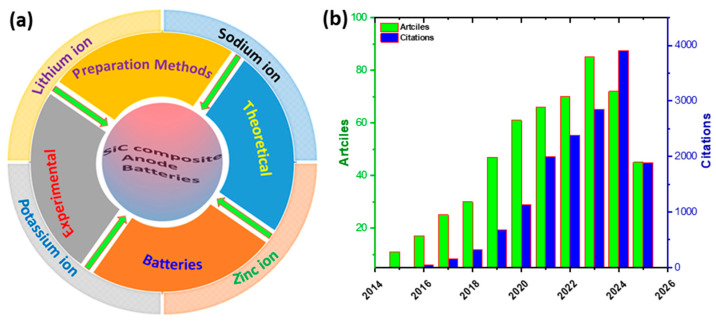
(**a**) Illustration of the main focus and content of this review. (**b**) The number of articles versus citations based on Web of Science and Scopus data using the keywords “SiC anode batteries” and “SiC composite batteries”.

The presented review highlights the controlled synthesis of silicon carbide composites for use as anodes for various batteries. The main preparation methods of SiC-based composites (i.e., ball milling, pyrolysis, chemical vapor deposition, and spray drying) are discussed (Figure 1a). The electrochemical performances of SiC composites as anodes for use in lithium-ion (LIBs), sodium-ion (NIBs), zinc-air (ZnBs), and potassium-ion batteries (PIBs) are discussed. The doping effect is comprehensively reviewed, both experimentally and theoretically. This is in addition to the current change and drawbacks for the utilization of SiC for rechargeable batteries. All the reported data in the presented review were collected from both Scopus and Web of Science without bias. Research dedicated to the synthesis of Si-based composites for batteries has grown significantly. This has become a hot topic, as shown in the exponential increase in publications (i.e., 1875 articles, cited ~46,025 times), including 529 articles on SiC anodes, cited 15,349 times (~29.01 citation/article) over the last decade (Figure 1b).

## 2. Fabrication Methods of SiC and Si/C Composites for Metal-Ion Batteries

There are various methods for synthesizing Si/C and SiC composites or composites for metal-ion batteries, which differ in their ability to tailor morphology, composition, and physicochemical properties. The main features and parameters, besides the pros and cons of each method, will be discussed in brief to address the challenges associated with SiC materials for use in batteries. This review will briefly discuss the preparation methods of SiC or Si/C composites because they are already discussed extensively in many prior reviews [55,56].

### 2.1. Ball Milling

**Figure 2:**
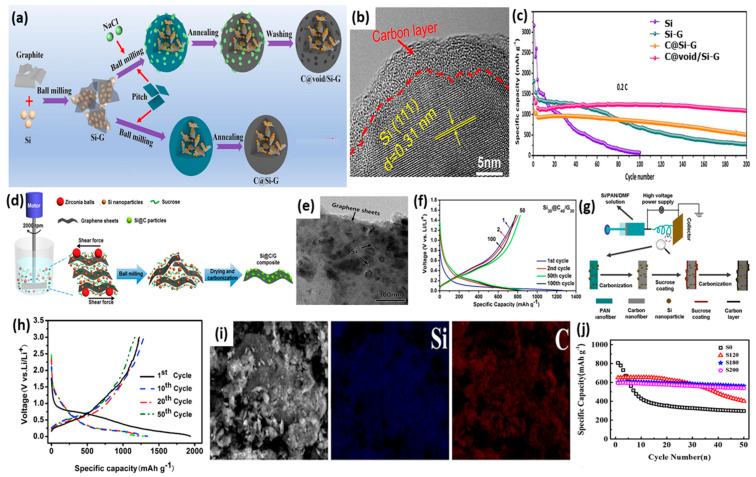
(**a**) The formation scheme and (**b**) TEM image of C@void/Si-g. (**c**) Cycling test of Si, Si-G, C@Si-G, and C@void/Si-G. Adapted with permission from Ref. [60] 2023, Elsevier. (**d**) The formation process, (**e**) TEM image, Si_30_@C_40_/G_30_ composite. (**f**) Galvanostatic charge/discharge profiles for Si_30_@C_40_/G_30_ composites after various cycles at 0.2 A/g. Adapted with permission from Ref. [61]. 2016, Elsevier. The schematic illustration of preparation (Si/C-CNFs-x) (**g**) and Galvanostatic charge/discharge profiles (**h**) for Si/C-CNFs-20 composites at 600 mAh/g. Adapted with permission from Ref. [73] 2015, Elsevier. (**i**) The SEM image and EDX mapping of SiC-Graphit-180 and (**j**) its cycling durability relative to Si/C-Graphite-x at 0.1 A/g. Adapted with permission from Ref. [76]. 2018, Springer.

The ball milling method is one of the mechanical methods used for the controlled synthesis of SiC; this occurs via the direct grinding of Si with C under a high-speed collision force using stainless steel or zirconia balls. This method is simple, productive, low-cost, facile, suitable for any powder precures, and feasible for large-scale application [57]. However, the inevitable aggregation of particles during milling along with the necessity for subsequent calcination are the main barriers. Various SiC materials and composites are prepared via ball milling in order to promote the activity and durability of metal-ion batteries [58,59]. For instance, Si undergoes ball milling with graphite to form SiG, then milling with pitch and NaCl as a template, followed by calcination and template removal to form C@void/Si-g as an anode for LIBs (Figure 2a) [60]. In this method, C@void/Si-g was coated uniformly with a carbon layer, and Si was centered in the core area (Figure 2b). C@void/Si-g was superior to C@Si-g without the template, and led to greater durability with a maintained capacity of 1082.7 mAh/g after 200 cycles at 0.2 C (Figure 2c); moreover, volume expansion was enhanced by nearly 41% after 500 cycles. This is owing to the isolation of Si from the electrolyte [60]. Si nanoparticles were mixed with expanded graphene and sucrose in an aqueous solution of water/ethanol via the wet ball milling method, then calcinated at 600 °C for 2h to the form Si_30_@C_40_/G_30_ composite for use as anodes in high-performance LIBs (Figure 2d,e) [61]. Si_30_@C_40_/G_30_ had a Li-storage capacity of 1259 mAh/g at 0.2 A/g in the initial cycle, besides a great high irreversible capacity with a nearly overlapping voltage profile after the 50th and 100th cycles (Figure 2f), indicating an increased reversible electrochemical reaction between Li ions and Si_30_@C_40_/G_30_. This is owing to the unavoidable formation of an SEI layer on Si and the outstanding surface area of the graphene nanosheets. The ball milling method is flexible, and can be combined with other methods in order to promote performance. SiC formed by ball milling alone showed a lower-capacity retention rate and reversible capacity [62,63], while both were boosted using the annealing method along with ball milling with calcination [64,65]. Si/graphite/multi-walled carbon nanotubes (MWNTs) [66] and nitrogen-doped carbon and Si composites were formed using polyvinylpyrrolidone (Si@C-2) [67]. The synthesis of Si/carbon nanotubes [68] was conducted in the same way, resulting in the formation of Si/C from citric acid along with Si [69]. Si/graphene anodes of LIBs formed via wet ball milling displayed a capacity of 850 mAh/g and greater capacity retention at (≈800 mAh/g at 5 A/g); this was superior to the capacity of anodes formed by dry ball milling, due to improved particle size uniformity and more intimate contact between them [70]. Si/SiC@C composites smaller than 1 μm in size were used as anodes for LIBs; these were prepared by ball milling f micro-Si and 6H-SiC, followed by coating them with a polydopamine layer then carbonization at 800 °C [71]. After 400 cycles, they exhibited a superior cycle performance of 814.6 mAh/g @ 1 A/g and a high capacity retention of 88.0%, as well as an excellent rate capability of 762 mAh/g at 5 A/g. This improved capacity is ascribed to the formation of the Si/6H-SiC heterostructure, which enhances charge transfer by accelerating Li^+^ diffusion via pseudocapacitive behavior. Moreover, the uniform distribution of inactive 6H-SiC mitigates mechanical stress and volume fluctuations in electrodes, contributing to the improved cycle stability of anodes [71].

### 2.2. Pyrolysis

Pyrolysis entails the thermal decomposition of organic materials under an inert atmosphere (i.e., N_2_, and Ar) to generate carbon with Si or coated Si particles. Pyrolysis is widely used in the synthesis of carbon materials from biomass wastes or other organic materials; it can be classified into carbonization, conventional pyrolysis, quick pyrolysis, flash pyrolysis (both liquid and gas), ultra-pyrolysis, vacuum pyrolysis, sequential pyrolysis, and methanation pyrolysis, according to the processing conditions (i.e., residence time, temperature, and heating rate) of the reactors [72]. The gases generated in situ from the decomposition of organic materials lead to the formation of a porous structure which is highly beneficial for the alleviation of the volume changes of Si during battery tests. Also, pyrolysis is compatible and usually combined with other methods such as sol–gel, electrospinning, template, etc. In the present study, Si/CNFs were formed via the electrospinning of Si/AN/DMF and then annealing, before being coated with carbon by annealing with sucrose to yield carbon-coated Si/C nanofibers (Si/C-CNFs-x) (x = carbon content) for use as anodes for LIBs (Figure 2g) [73]. The carbon coating content was adjusted using different concertation of sucrose as the carbon source, which controlled the supercapacitor performance. Si/C-CNFs-x outperformed CNFs and Si/CNFs, but Si/C-CNFs-20 was the optimum, with a capacity of 350 mAh/g at 5000 mA/g; moreover, it maintained a discharge capacity of 1215.2 mAh/g after 50 cycles (Figure 2h). Carbon coating alleviates Si volume expansion, preserves structural durability, and provides a pathway for Li-ion mobility, leading to enhanced performance. The same was observed when Si/C composites were formed via the calcination of Si and flake graphite with phenolic resins [74]. Also, Si nanoparticles which were annealed with phenolic resin to form Si/C composites for use as anodes in LIBs showed a reversible capacity of 400 mAh/g, along with great cycle performance [75].

Si/C-Graphite-x composites were prepared by the liquid solidification of Si nanoparticles, graphite, and coal tar pitch, as well as heat treatment at different temperatures (x = 120, 180, and 200) under vacuum followed by pyrolysis at 850 °C for 3 h under Ar [76]. SiC-Graphit-180, which is used as an anode for LIBs, has an amorphous and irregular shape, but with good distribution of Si and C carbon layers (Figure 2i). SiC-Graphit-180 showed the best performance with a reversible capacity of 602.4 mAh/g, charge–discharge efficiency of 82.3%, and high cycling durability, with a capacity retention of 93.4% at 0.1 A/g after 50 cycles (Figure 2j) [76]. Graphite enhances the dispersion and contact between Si and C derived from coal tar pitch, and increases electronic conductivity; meanwhile, the amorphous C structure acts as a separator between C and graphite that buffers Si volume expansion and boosts the conductivity between Si and graphite. Si/C-Graphite-180 showed a superior battery performance than others such as Si/graphite/carbon nanotubes@C composites [77] and graphite/silicon/pyrolyzed-carbon composites [78]. This indicates the significant effect of pyrolysis conditions on promoting storage capacity and durability.

Thereby, Si/C-x was doped in situ with N and O (x = 250, 300, 350, and 400 °C) for use as an anode for LIBs; this formed Si nanoparticles and polyacrylonitrile via oxidative pyrolysis, showing that performance depended on pyrolysis temperature (x = 250, 300, 350, and 400 °C). However, SiC-400 was found to be the optimum, with a discharge capacity of 1555 mAh/g at the 100th cycle, and a lithiation capacity close to 4000 mAh/g, which is superior to the capacity of Si combined with 3.75 Li ions (Li_15_Si_4_) at 3579 mAh/g [79]. These findings may allow the synthesis of Si/C doped with heteroatoms, which is a facile method of synthesizing Si-based composites doped in situ with non-metal atoms from various polymers. The same was observed for Si/C@NGs composites formed from the pyrolysis of Si nanoparticles with graphite, polyvinylpyrrolidone, and carboxymethyl cellulose sodium salt [80].

**Figure 3:**
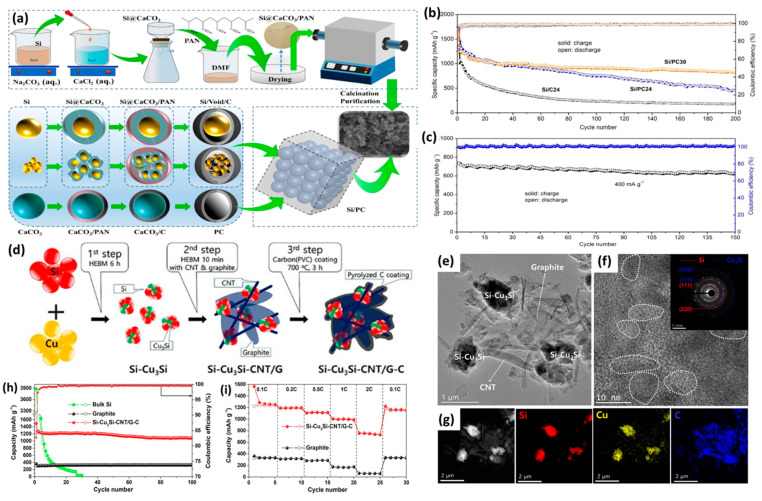
(**a**) The formation scheme of Si/PC-x and proposed structures of other shapes. (**b**) Cycling durability at 0.2 A/g of Si/PC-30 compared with Si/PC-x. (**c**) Cycling durability at 0.4 A/g after the rate test for Si/PC-30. Adapted with permission from Ref. [81]. 2021, Elsevier. (**d**) Schematic illustration of the formation, (**e**) TEM image, (**f**) HRTEM image, and (**g**) scanning TEM with EDX mapping of Si-Cu3Si-CNT/G-C. (h) Cycling stability of Si-Cu3Si-CNT/G-C at 0.2 A/g relative to graphite at 0.1 A/g, and bulk Si at 0.2 A/g. (i) Rate capabilities of graphite at 0.3 A/g and Si-Cu3Si-CNT/G-C at 1.2 A/g. Adapted with permission from Ref. [82]. 2020, Elsevier.

Micro-/nano-silicon nanoparticles were encapsulated with N-doped porous carbon (PC), using CaCO_3_ as a template, and polyacrylonitrile (PAN) as a C/N source (Si/PC-x) (Figure 3a) [81]. This facile method forms Si@PC, which is composed of three morphologies, including two yolk–shell-structured Si@void/C and Si_n_@void/C (granadilla-like) composites, and porous carbon Si@C and Si_n_@C composites. This was formed without a hollow space between the Si core and the carbon shell when isolated or aggregated Si particles were not sufficiently coated with CaCO_3_. The stability and rate performance of the anodes obtained (Si/PC-x) for use in LIBs were (X = PAN content)-dependent; however, Si/PC-30 was found to be the optimum, maintaining 830 mAh/g after being discharged/charged at 200 mA/g for 200 cycles with a retention of 81.5% (Figure 3b,c) [81]. This is due to its unique porous honeycomb structure and improved electrical conductivity due to the N-doped carbon.

New conditions for the formation of SiC composites are yet to be explored for the promotion of LIB performance. In response, recently, porous Si was formed from Cu_3_Si via ball milling. This then underwent pyrolysis to improve the thermal reduction approach; it was then pyrolyzed to allow for carbon coating with various carbon materials, including carbon black (Si-Cu_3_Si-CB), graphite (Si-Cu_3_Si-G), carbon nanotube (Si-Cu_3_Si-CNT), and carbon nanotube/graphite (Si-Cu_3_Si-CNT/G) (Figure 3d) [82]. Si-Cu_3_Si-CNT/G displayed uniform distribution of Si (~10 nm size) with Cu_3_Si nanoparticles, which are coated with interconnected 1D-CNT and 3D graphite (Figure 3e,f). This is also seen in the scanning TEM and EDX mapping that confirms the even distribution of Si and Cu_3_Si coated with CNT and G (Figure 3g). The activity of Si_x_-Cu_x_-Si depended on the Si/Cu alloy ratio, but Si_x_-Cu_3_-Si was found to be optimum. Meanwhile, Si-Cu_3_Si-CNT/G coated with carbon via annealing with polyvinyl chloride (Si-Cu_3_Si-CNT/G-C composite) showed the highest performance, with a reversible capacity of 1237 mAh/g, coulombic efficiency of 82.8%, durability over 100 cycles, and an excellent rate capability of ~1000 mAh/g at 1C (Figure 3h) [82]. This indicates the commercial feasibility of Si-Cu_3_Si-CNT/G-C due to the well-dispersed Si and Cu_3_Si nanocrystals (Figure 3i) acting as a Li-inactive conducting matrix within the multiple interconnected C-matrices [82]. Although pyrolysis is simple and effective, its high energy demand and need for safety precautions remain; moreover, the lack of controlled size distribution presents a challenge. However, coupling annealing with other methods led to significant improvements.

Si/SiC/C nanocomposite microspheres 2 μm in size were prepared using a mixture of TEOS, 3-glycidyloxypropyltrimethoxysilane, resorcinol, and formaldehyde via the sol–gel method. Then, the formed aerogel was calcinated at different temperatures, followed by magnesiothermic reduction; this was used as an anode for use in LIBs [83]. Calcination at 390 °C demonstrates the best electrochemical performance; it has a reversible capacity of 1513 mAh/g at a current density 0.1 A/g, and coulombic efficiency of 79.1% with a high-capacity retention rate after 200 cycles of 87.8%. This is related to the optimum C content in composites: 65.0 wt% Si, 16.8 wt% SiC, and 18.2 wt% C. Porous 3D graphene aerogels (Si/GA) were prepared via the facile hydrothermal synthesis of Si/GO aerogels; this was followed by freeze-drying and then reduction at 1000 °C under the Ar/H_2_ mixture [84]. This displayed good cyclic stability with a specific capacity of 850 mAh/g after 200 cycles and a coulombic efficiency of 99% at a current density of 0.5 A/g. The exceptional performance of graphene aerogels, due to their porous architecture, promotes electrolyte penetration, improves Li-ion diffusion, and reduces the volumetric expansion of silicon particles, therefore enhancing rate capability and cycle stability during delithiation processes. A plasma-assisted discharge procedure was utilized to attach nano-sized Si particles in methanol, followed by quick quenching and a subsequent sintering phase at a high temperature of 1100 °C under an Ar environment to produce a Si/SiC/C composite (M-Si) [85]. The resultant M-Si electrode exhibited a reversible specific capacity of 563 mAh/g after 100 cycles at 0.1 C, with a coulombic efficiency of 99%, surpassing bare Si electrodes. The improved performance is ascribed to the composite construction, which enhances electrolyte penetration, promotes Li^+^ diffusion, and efficiently mitigates the volume expansion of silicon during cycling.

### 2.3. Spray Drying

**Figure 4:**
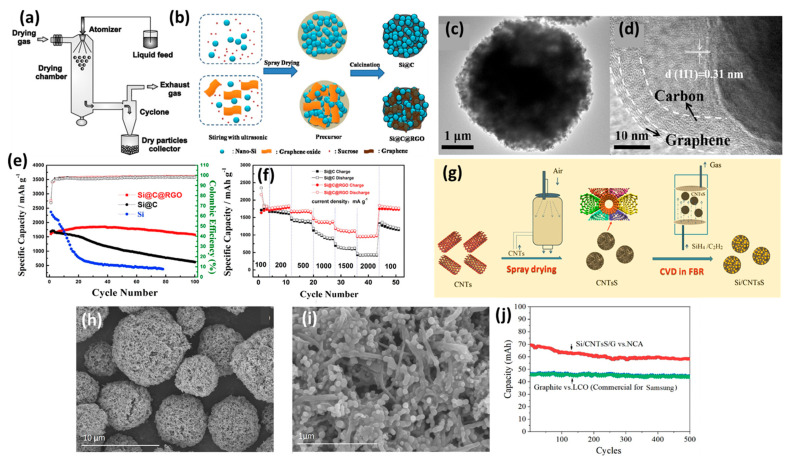
(**a**) Illustration of the spray-drying process. Adapted with permission from Ref. [87] 2015, Elsevier. (**b**) Schematic of the preparation process of Si@C@RGO, its TEM image (c), and (d) its HRTEM image. (e) Cyclic profile of Si, Si@C, and Si@C@RGO composites at 100 mA/g in the 1st three cycles and then at 0.2 A/g. (f) Rate performance of Si@C and Si@C@RGO composites under various currents. Adapted with permission from Ref. [91] 2017, Elsevier. Schematic illustration of the synthesis of Si/CNTs (**g**) and its (**h**) TEM image and (**i**) HRTEM image. (**j**) The capacity of pouch cells with a commercial graphite anode and a LiCoO_2_ (LCO) cathode compared with commercial Samsung LIBs. Adapted with permission from Ref. [96] 2021, Elsevier.

Spray drying is one of the main approaches used for the production of dry powder; this is carried out via the quick evaporation of a liquid solution by heating with gas at an elevated temperature [86]. Mainly, the fluid is fed into the drying chamber via a peristaltic pump through an atomizer (i.e., rotary, pressure, or two-fluid nozzle) to induce atomization by centrifugal pressure, and kinetic energy, correspondingly) [87]. The small droplets obtained are promptly evaporated, forming dry particles which are isolated from the drying gas via deposition in a glass collector by a cyclone (Figure 4a). SiC composites prepared by spray drying offer various advantages, like quick drying, powder yield, uniform particle size, and feasibility for practical applications; their high energy demand, high cost, and complex process conditions are the main disadvantages [87]. The high surface tension acting on the solution during the spray-drying stage drives the formation of spherical-like morphologies. Thus, various SiC composites were synthesized via spray drying; these composites varied in terms of their electrochemical performance. Spherical-like SiC composites were formed by spray drying, followed by the annealing of Si nanoparticles, glucose, and graphite; these exhibited a reversible capacity of 602.7 mAh/g and coulombic efficiency of 69.71%, with a good cyclability similar to that of commercial graphite [88].

S/C composites formed by spray drying and annealing, using Si nanoparticles and citric acid as a carbon source, demonstrated that performance depended on the carbon content; however, one composite with a low content of 5.6 wt% performed best, having a capacity of 1860 mAh/g after 60 cycles at 100 mA/g [89]. This is due to the coating of Si nanocrystals with floc-like C layers and fragments, precluding Si expansion and improving electrical conductivity. Si/C composites obtained via spray drying Si nanoparticles with polyvinyl alcohol as a C-source demonstrated enhanced performance which was slightly relative to that of pristine Si; however, the addition of a cross-linker led to a significant improvement [90]. Without a cross-linker, the contact between C and Si increased, but with a cross-linker, the solubility of the polymer increased; furthermore, the contact between C and Si was reduced, leading to improved conductivity and cycle performance for Si nanoparticles [90]. These studies did not demonstrate a substantial improvement in the electrochemical performance of Si/C anodes for use in LIBs when compared with others. Thus, as found in other methods like annealing and ball milling, adding an additional C-layer or an additive, or altering the reaction conditions, is mandatory. In this context, Si/C/rGO anodes for use in LIBs were formed via spray drying and the subsequent annealing of Si, carbon, and graphene oxide; Figure 4b shows the formation of Si nanoparticles coated with a C-middle layer and RGO outer layer (Figure 4c,d) [91]. Si/C/rGO displayed a superior discharge-specific capacitance (2124 mAh/g) and initial coulombic efficiency (75.3%) than Si and Si/C, alongside a greater capacity retention of (94.9%) after the 100th cycle at 200 mA/g (Figure 4e,f). This is due to the addition of rGO, which enhanced the conductivity, reduced the connate of Si to the electrolyte, and alleviated Si volume expansion [91]. Si nanoparticles synthesized using the radio frequency induction plasma were then spray dried and annealed with graphite and citric acid to form graphite@plasma nano-silicon@carbon (AG@PNSi@C); this displayed a discharge/recharge capacity of 553/448 mAh/g, with a maintained durability for 500 cycles and a high resistance against high currents [92]. Graphite enhances the electrical conductivity, and plasma Si nanoparticles enhance the specific capacity.

Although rGO enhanced the performance, it is still ambiguous as to whether graphene or C performs better as a coating layer for Si. In response, to check whether C or graphene coating is better [93], Si/C composites were prepared by spray drying Si with graphene then annealing, which showed electrochemical performance depending on the ratios Si/graphene; however, a ratio of ¼ was demonstrated the best results, with initial specific discharge/charge capacities of 2869.9/1298.1 mAh/g, respectively; this ratio also demonstrated a better rate capability even at 1000 mA/g. Although the capacity increases with decreases in the graphene content, the effects of carbon-coated Si should be compared with the effects of graphene-coated Si to underline the real effect. Coupling spray drying with the electrostatic self-assembly method generates 3D spherical-like silicon/graphite (Si/G) anodes for use in LIBs; performance varied depending on the method of spray drying, including single (SD1-Si/G) and double (SD2-Si/G) [94]. The as-made SD2-Si/G anode was the best; this demonstrated an initial discharge-specific capacity of 1020 mAh/g and good cycling durability at 400 mA/g, owing to the precluding Si-volume change, as evidenced via the in situ dilatometry. Si/C anodes used in LIBs are composed of Si-embedded graphite in a core–shell structure, along with C layers. The shell is formed by spray drying along with the C-source cladding method performed twice; the results demonstrated a reversible capacity and initial coulombic efficiency of 936.4 mAh/g and 88.6%, respectively, with a capacity retention of 80% after 680 cycles [95]. This structure is useful for controlling Si-volume expansion, as well as enhancing electrical conductivity and electrochemical performance. However, further improvements are needed in order to successfully utilize spray drying for the controlled synthesis of Si/C composites as anodes for use in LIBs; moreover, more carbon layers should be used in order to enhance performance.

### 2.4. Chemical Vapor Deposition (CVD)

The CVD method is one of the most efficient approaches for ensuring the effective growth of SiC and other materials, resulting in well-defined morphologies and composition. In addition, it can be used to tailor physiochemical merits (i.e., catalytic and electronic) for various applications [97]. CVD entails the direct use of gaseous reactants in a tube furnace, which promptly decompose to form atomic species which are subsequently deposited on the substrate. CVD was initially used for the synthesis of graphite-carbon-coated Si and carbon nanotubes; however, later, it was used for the deposition of Si using precursors like SiH_4_ or SiHCl_3_ [98]. The temperature, time, and gas flow rate are the main factors tailored to the reaction kinetics and, consequently, the structured atomic arrangement during crystal growth. At high temperatures and gas flow rates, the deposition yield increases, but the resistance may increase; thus, the performance may be affected. Various Si/C anodes for use in LIBs were synthesized by CVD; these varied in terms of their performance, driven by CVD conditions and parameters.

Si/multi-walled carbon nanotube (MWNT) (Si/MWNT) composites were formed via a double-step CVD process using xylene (C_8_H_10_) as the C source and (Fe(C_5_H_5_)_2_) as the catalyst to form MWNTs; this was then followed by CVD using silane gas to form Si/MWNTs to be used as anodes in LIBs [99]. The as-made Si/MWNT anode displayed a reversible stable capacity of 2049 mAh/g, with only 19.7% capacity loss. This is due to well-aligned tethered MWNTs and the homogenous distribution of Si nanoparticles; moreover, their inner spacing lead to reversible alloying/dealloying with Li, leading to a negligible loss of contact with MWNTs [99]. This study demonstrates the significant effect of 1D carbon support on promoting the performance of Si anodes. Core–shell nanoparticles of Si and CNTs formed by CVD demonstrated high capacity at 1496 mAh/g at 0.1 A/g, maintaining a cycling stability of 80% after 300 cycles. This was attributed to the three-dimensional conductive networks f CNT-coated Si [100]. Si/CNT microspheres were initially prepared using spray drying to form CNTs, which were used as supports and as in situ hard templates to support the deposition of Si nanoparticles via CVD in fluidized bed reactors (FBR) (Figure 4g) [96]. Si/CNTs are composed of porous microspheres and Si nanoparticles enclosed in CNTs (Figure 4h,i). Si/CNT anodes demonstrated a specific capacity of over 500 mAh/g, with the capacity of the pouch cell proving to be nearly 1.3 times higher than commercial Samsung LIBs (Figure 4j). This study supports the synthesis of Si contained in CNTs through CVD in FBR, which eliminates the cons of low production yield and time lost in CVD [96]. Meanwhile, the encapsulation of Si with CNTs prevents Si volume expansion and enhances electrical conductivity, along with stabilizing Si against aggregation or separation during the charge/discharge cycles [96].

**Figure 5:**
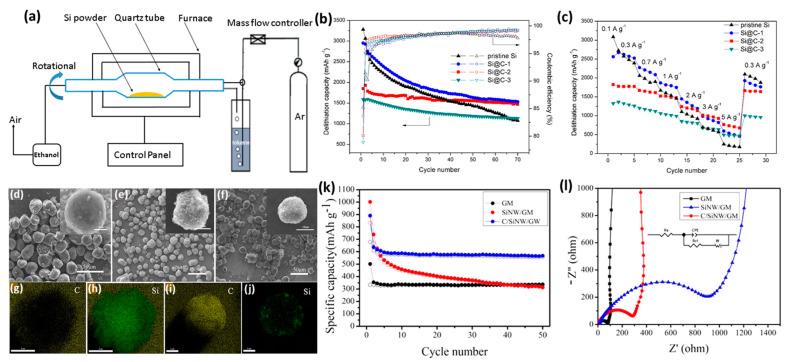
(**a**) Diagram for the rotational CVD carbon system for the preparation of Si@C-x. (**b**) Cycling durability of Si@C-x at 0.3 A/g, showing the 1st two cycles at 0.1 A/g and their delithiation capacities (**c**) at various currents. Adapted with permission from Ref. [104] 2014, American chemical society (ACS). SEM images of (**d**) SiNW/GM and (**e**,**f**) C/SiNW/GM. EDX elemental mapping of (**g**,**h**) SiNW/GM and (**i**,**j**) C/SiNW/GM (**k**) Cycling performance of GM, NW/GM, and C/SiNW/GM at a current density of 0.2 C over 50 cycles (**l**) Nyquist plots of GM, SiNW/GM, and C/SiNW/GM. Adapted with permission from Ref. [105] 2021, ACS.

Likewise, Si nanoparticles were used to encapsulate carbon nanofibers (CNFs) via the electrospinning of PAN fibers using the electrospinning and annealing method to form Si-x@CNF (X = Si content 10, 30, and 50 wt%). This was then coated with a carbon layer via CVD, using acetylene (C_2_H_2_) to produce Si@CNF-C-x (x = CVD times 30, 60, and 90 min) [101] through the thermal decomposition of the carbon precursor gas. Si-x@CNF-C anodes demonstrated better performance than Si@CNF, but Si-30@CNF-C-90 proved optimum with over 200% improvement in cycling performance, owing to the prevention of Si leaching or volume expansion [101]. This study indicates the effect of carbon coating and CVD time on enhancing the performance of SiC anodes for use in LIBs. However, C-coated Si is not uniform when using traditional CVD, which could be solved by repeating CVD to improve the uniformity of the coating [102]; however, this is complicated and time-consuming. Another solution was using pressure-pulsed CVD that can ease the penetration of gas to Si via repeated evacuation and gas feed [103]. Another achievement is the utilization of rotational CVD coating (Figure 5a) that allows for the initial rotation of Si nanoparticles inside the rotated quartz tube furnace under the following conditions: the compression of toluene plus argon to form the carbon source, carried out at a temperature of 800 °C, repeated at different times to form Si@C-x (x = 1, 2, and 3 h) [104]. The Si@C-2 anode displayed a carbon layer thickness of 12 nm; this anode delivered a specific capacity of 1600 mAh/g at 0.3 A/g for 70 cycles, and with a miniated capacity of 750 mAh/g at 5 A/g, which was nearly 3 times higher than that of bare Si nanoparticles (Figure 5b). It was also stable under all tested current densities (Figure 5c). This is due to the uniform carbon layer of coated Si. Rotational CVD allows for uniform carbon coating with tunable thickness; this is also feasible for various substances such as acetylene.

Thus, the preparation conditions and nature of CVD are crucial factors in the synthesis of SIC anodes for use in LIBs. Thus, the effect of deposition temperature must be considered. Changing the deposition temperature resulted in the formation of SiC-coated C/C wires with different diameters [106]; meanwhile, the temperature had a substantial effect on the activation energy and structural growth of SiC [107]. These studies used solid Si nanoparticles with low surface areas; thus, due to their high surface areas and accessible active sites, porous nanostructures can enhance the performance of LIBs. In response, Si-SiOx (pSi) composites consisting of Si, SiO, and SiO_2_ were initially formed via a disproportionation process. These composites were then coated with a carbon nanofiber shell (pSi-CNFs-x) via CVD in the presence of an Fe-Ni catalyst at different times (x = 0.5, 1, and 2 h), making them suitable for use as anodes for in LIBs [108]. The outcome mainly depended on CVD time, but pSi-CNF-0.5 proved best with a reversible capacity of 1411 mAh/g and capacity retention of 74% after 100 cycles at 0.2 A/g;^,^ moreover, it demonstrated a sustained reversible capacity of 735 mAh/g at 1 A/g after 300 cycles, along with a capacity retention of 86%. This is due to its porosity and uniform carbon layer, which enhance its electrical conductivity and preclude Si volume expansion. The carbon/silicon nanowire/graphite microsphere (C/SiNW/GM) was formed via CVD. This entailed the use of a freeze-dried graphite microsphere with a Ni precursor as the catalyst, along with chloromethylsilane as the Si/N source; this was placed in a rotating furnace at 800 °C and followed by washing to remove Ni. The resulting SiNW/GM composite was placed in the rotational CVD system, with acetylene as the carbon precursor, in order to yield C/SiNW/GM composites [105]. SiNW/GM has a rough surface, with uniform distribution of Si nanoparticles (Figure 5d), while the C/SiNW/GM anode has a smother surface (Figure 5e). SiNW/GM comprises curved and interplayed Si nanowires grown on the GM microsphere (Figure 5f). EDX mapping displayed lower uniformity when carbon coating SiNW/GM (Figure 5g,h) than for C/SiNW/GM, which had uniform carbon coating (Figure 5i,j) [105].

As an anode used in LIBs, the performance of C/SiNW/GM was superior to that of SiNW/GM and GM, with an initial charge/discharge capacity of 678.2/890.1 mAh/g, a reversible capacity of 585 mAh/g after 50 cycles, and a coulombic efficiency of 98.3% (Figure 5k). This is due to carbon coating, which reduces the charge transfer resistance significantly, as shown in the Nyquist plots (Figure 5l). C/SiNW/GM showed better performance than the amorphous Si/graphite composite [109], Si/edge-site-activated graphite [110], and Si-embedded graphite/carbon [111] formed by via CVD. Despite the achievements noted in the utilization of CVD for the controlled synthesis of SiC composites as anodes for batteries, further efforts are still needed to simplify the process, enhance the production yield, and increase the anode performance (i.e., long life cycle, specific capacity, and energy density).

## 3. Doped SiC Anodes for Recharge Batteries

### 3.1. Theoretical Studies of Doped SiC Anodes

**Figure 6:**
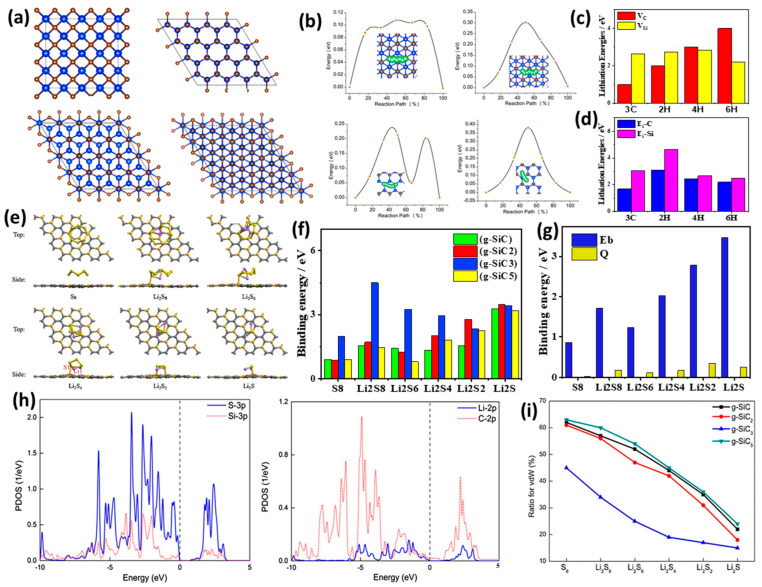
(**a**) Molecular structure of bulk SiC materials. (**b**) Calculated Li diffusion barriers (eV) on 3C (111), 3C ($\bar{111}$), 2H (0001), and 2H (000$\bar{1}$) surfaces with the schematic representation of corresponding diffusion paths. (**c**) Formation energies (eV) of VC and VSi. (**d**) Lithiation energies (eV) at the most favorable silicon vacancy and carbon vacancy sites for different polytypes of silicon carbide by GGA-PBE. Adapted with permission from Ref. [112] 2020, *Phys. Chem. C*. (**e**) The most stable adsorption configurations of various S-containing species adsorption on g-SiC_2_. (**f**) A comparison of the anchoring effect of g-SiC_2_ with different siligraphene. (**g**) The binding energies (Eb, eV), and charge transfer (Q, |e|) of various S-containing species on g-SiC_2_. (**h**) The computed projected density of states (PDOSs) of S (of Li_2_S_4_ and Si of g-SiC_2_) and Li (of Li_2_S_4_ and C of g-SiC_2_). (**i**) Ratio for vdW interaction for the adsorption of S-containing species on various siligraphenes. Adapted with permission from Ref. [123] 2018, *Appl. Surf. Sci*.

DFT calculation is an accurate and efficient method for investigating challenging property measurements, such as the energy storage capacity of Si/C and other materials, while also predicting their performance as anodes/cathodes for use in batteries. This is achieved by calculating the binding energy, charge flow during lithiation and delithiation, and energy barriers. For example, the lithiation capabilities of SiC as an anode were evaluated based on both surface and bulk properties, including bulk 3C, 2H, 4H, and 6H-SiC, with and without defects, and various surface configurations (Figure 6a) [112]. In the case of surface-doped SiC, the lithiation energy was significantly negative, indicating that lithiation was feasible. The calculated lithium diffusion barriers exhibited the following order: 3C (111) < 2H (0001) < 3C (111) < 2H (0001); this is illustrated in Figure 6b. This variation arose from differences in the stacking orders of the layers beneath the top surface of SiC, leading to distinct lithiation energies and lithium diffusion barriers at low lithium concentrations. It is important to note that charge transfer is largely independent of the binding energy and lithium-ion storage. Despite this study providing systematic calculations for the lithiation capacity and charge transfer in both bulk and surface SiC, with and without B vacancies, the observed capacity remains quite low and impractical for real-world applications. This limitation stems from the poor electrochemical properties of SiC, which increase the charge transfer resistance of lithium to carbon and silicon. A potential solution is the incorporation of a conductive material with SiC, prompting the exploration of graphitic SiC as an anode for lithium-ion batteries (LIBs). The calculated lithiation energies for bulk 3C, 2H, 4H, and 6H-SiC were found to be positive, indicating energetically unfavorable conditions. This finding aligns with the inferior electrochemical properties of SiC, which are consistent with experimental data reported elsewhere [113,114]. The introduction of surface vacancies resulted in a decrease in lithiation energies for a single lithium atom across all materials, yielding negative values in the order of 2H < 4H < 6H < 3C in terms of V_Si_, and 6H < 3C < 2H < 4H for V_C_ (Figure 6c). Additionally, the highest lithiation energy observed for Li-C is attributed to the strong repulsion between lithium and silicon, along with their minimal distance apart. Conversely, carbon, with its higher electronegativity compared to silicon, slightly mitigates the lithiation energy (Figure 6d). Furthermore, the charge of lithium in VSi is lower than that of lithium in SiC without vacancies, allowing for the possible conversion of V_Si_ to V_C_, where 3C-SiC with defects of V_C_ + C_Si_ outperforms one with a single V_Si_. However, positive lithiation energy may still be influenced by repulsion from silicon and C_Si_. In the case of B-doped bulk SiC, CM5 charges transfer from lithium to boron, carbon, and silicon after lithiation, and this doping can shift the lithiation energy from positive to negative due to boron’s smaller atomic radius and lower electronegativity compared to silicon.

Ab initio analysis of boron-doped armchair silicon carbide nanoribbon (B-ASiCNR) for use as an anode material for LIBs demonstrates superior storage capacity at 836 mAh/g, outperforming the B-C Metal Sheet at 485 mAh/g and BAGNR at 783 mAh/g [115]. The B-doped hexagon of the substrate is identified as the most favorable site for the adsorption of Li atoms, with an adsorption energy of −3.76 eV. This value exceeds the adsorption energy of the ASiCNR, which is −1.25 eV for Li atoms at the same site. Consequently, B-ASiCNR forms stronger bonds with Li atoms than pristine ASiCNR, making it a significant candidate for anode material in LIBs. DFT studies utilizing the Quantum ESPRESSO package on zinc blende 3C-silicon carbide (3C-SiC) as an anode material for LIBs [116] reveal enhanced lithium intercalation efficiency. This is calculated using the pseudopotential plane-wave method, which introduces a Si monovacancy to facilitate Li intercalation. In the case of Li_4_Si_31_C_32_, the defected Si_31_C_32_ structure becomes energetically favorable, exhibiting a Li capacity of 86 mAh/g and an average intercalation voltage of 0.81 V. This aligns with the known properties of anode materials; these are exothermic and energetically favorable for accommodating up to six Li atoms, in contrast to pristine 3C-SiC, which is thermodynamically unfavorable. N-doped silicon carbides (SiCNTs) analyzed using spin-unrestricted DFT show the potential of N-doping for promoting the adsorption and diffusion of Li in LIBs [117]—in particular, N@Si (N replacing Si), which presents a superior binding energy of −2.36 eV compared to N@C (N replacing C) and pristine SiCNTs, both at −1.47 eV. The calculated energy barrier of SiCNT was about 10.23 eV, which means that it is virtually impossible for Li to diffuse through the hexagons of CNT. Meanwhile, after N-doping, the energy barriers on N@Si- and N@C-doped SiCNTs were decreased to 8.24 and 6.23 eV, respectively. This indicates the possible effect of N-doping at Si and C sites on decreasing the energy barrier of Li-diffusion, but the energy barrier is still high and can prohibit Li diffusion through the walls of N-doped SiCNTs [117]. The introduction of lithium into a silicon layer supported by nitrogen-doped silicon carbide at various lithium-to-silicon ratios (1:1, 2:1, and 3:1) improves energy and structural stability in LIBs through quantum-chemical modeling, using density functional theory [118]. The absorption energy for the Li:Si ratio of 1:1 is 2.05 eV, and the reduced thickness of the absorbing layer results in a stable structure, as Li atoms are uniformly distributed within the Si layer and in contact with the SiC support. This configuration surpasses the ratios of 2:1 and 3:1, which yield absorption energies of 1.98 eV and 1.90 eV, respectively, leading to structural breakdown. Phonon spectrum calculations and ab initio molecular dynamics (AIMD) simulations were conducted through a targeted structure search of T-C_2_Si as an anode for LIBs; this material exhibits superior X-ray diffraction (XRD), fitting with experimental results [119]. This material outperforms traditional anode materials such as graphite and pure Si, showcasing a high specific capacity of 515 mAh/g, an average open-circuit voltage of 1.14 V, and a minimal volume change of 1.6% during charging and discharging. These characteristics are attributed to metallic features within the band structure that contribute to high intrinsic electronic conductivity. Furthermore, DFT indicates that penta-siligraphene (P-Si_2_C_4_) is anticipated to provide a high storage capacity of 1028.7 mAh/g, along with a superior band structure. The calculations of T-C_2_Si reaffirm its high specific capacity of 515 mAh/g, an average open-circuit voltage of 1.14 V, and a minimal volume change of 1.6% during charge and discharge cycles. The metallic features in its band structure play a crucial role in its high intrinsic electronic conductivity. In the same vein, DFT calculations reveal that P-Si_2_C_4_, when used as an anode material for LIBs, is projected to achieve a storage capacity of 1028.7 mAh/g with a superior band structure in comparison to penta-graphene (3.46 eV) [120], as determined using the Heyd–Scuseria–Ernzerhof (HSE06) hybrid functional method (2.35 eV). This configuration enables rapid charge and discharge due to low lithium migration energy barriers, and ensures dynamic stability at elevated temperatures (2000 K). Additionally, DFT computations for 2D silicon carbide (SiC) layers predict a higher storage capacity of 699 mAh/g [121], which is three times greater than that of graphite. The low diffusion barrier of 0.40 eV indicates good rate capability, while the layered structure facilitates efficient charge transfer, enhancing electronic conductivity compared to conventional graphite anodes.

First-principle calculations of SiC layers as a Li intercalation compound indicate that the lithiated structure exhibits greater stability, as the intercalation energy becomes more negative with increasing lithium concentrations. This performance surpasses that of bulk SiC structures, such as zinc blende and wurtzite [122]. The ionic diffusion shows an activation energy of 0.046 eV, and the diffusion coefficient is of the order of 10^−11^ m^2^/s. The intercalation voltage is 1.85 V. ReaxFF simulations suggest that volume expansion in SiC layers is lower than that in silicon (Si). When considering armchair silicon carbide nanoribbon (ASiCNR) as a potential anode material for use in LIBs, the density functional theory (DFT) approach predicts an open-circuit voltage with a minimum value of 1.15 V and a maximum storage capacity of 818 mAh/g, with the adsorption energy of ASiCNR increasing as more lithium atoms are intercalated spontaneously [115].

DFT predictions of the adsorption configurations for S species on g-SiC molecules in lithium-sulfur batteries (LSIBs) indicate that S-containing molecules are preferentially localized either parallel or perpendicular to the surface of g-SiC_2_ (Figure 6e) [123]. All potential adsorption sites were examined, revealing that the S8 cluster is thermodynamically favorably adsorbed in a parallel orientation to g-SiC_2_, suggesting favorable physical bonding over chemical bonding. When comparing the anchoring effects of g-SiC_2_ with various structures (Figure 6f), researchers found that g-SiC is similar to g-SiC_2_, exhibiting moderate binding strength but a wider band gap, which may limit its effectiveness as an electrode material. For g-SiC_3_, while high binding energies (>2.00 eV) are observed with S-containing species, these lead to the decomposition of Li_2_Sn clusters, rendering them less suitable for use as anchoring material. Although g-SiC_5_ demonstrates moderate binding strength, its lack of experimental availability restricts practical application. g-SiC_2_ strikes a balance between binding strength and structural integrity. It is noted that as the concentration of S-containing species increases, the Li-S and S-S bonds strengthen, while charge transfer diminishes (Figure 6g). Hirshfeld analysis indicates that charge transfer in g-SiC_2_ occurs from Si to C. The interaction strength between optimized g-SiC_2_ and Li_2_Sn suggests a stronger interaction with increased lithiation; the binding energies of soluble Li_2_S_8_, Li_2_S_6_, and Li_2_S_4_ are significantly higher than those of commonly used electrolyte solvents (e.g., DME and DOL). According to the criteria proposed by Zhang et al., g-SiC_2_ serves as a moderate anchoring material capable of immobilizing soluble Li_2_Sn species and preventing their dissolution into solvents [124]. The high stability of the intact structure of soluble species is assessed by the negative energy difference between the decomposed structures and the intact Li_2_Sn cluster. The partial density of states (PDOS) of the adsorption systems for soluble Li_2_Sn species was observed to be near the level of Fermi energy (Figure 6h). g-SiC_2_ retained its semiconducting properties following the adsorption of Li_2_Sn species; this was attributed to a slight increase in the VBM, which readily provides electrons for redox reactions involving the immobilized Li_2_Sn species, as indicated by the GGA/PBE method. Regarding van der Waals (vdW) interactions, all siligraphenes exhibited significant contributions to the adsorption of S_8_; specifically, g-SiC_3_ demonstrated a strong vdW interaction, while g-SiC and g-SiC_5_ showed moderate vdW interactions (Figure 6i). The impact of these vdW interactions can significantly influence the adsorption of Li_2_Sn species on anchoring materials, which is crucial for understanding their effects on LSIBs.

Sulfur was incorporated into CNTs/SiC to create S-CNTs/SiC composites; these were successfully prepared using catalytic chemical vapor deposition (CCVD) and serve as cathodes for LSIBs. They exhibit a superior cycling stability, which comes from the growth of carbon nanotubes on the SiC matrix [120]. The composites achieved high levels of performance, featuring a S content of 75.6 wt% and a discharge capacity of 685 mAh/g at a rate of 0.1 C after 100 cycles. They maintained a capacity of 316 mAh/g after 400 cycles at 1C, providing significant storage capacity and reducing the loss of active substances. This reduction is attributed to a combination of physical and chemical adsorption mechanisms that effectively minimize active substance loss during cycling. In contrast, S-SiC electrodes, which directly interact with lithium polysulfides through Si-S bonding, demonstrate lower specific capacities, as verified by DFT calculations. Additionally, particle swarm optimization combined with DFT studies reveals that three-dimensional microporous SiC_4_ crystals show potential as anode materials for NIBs [125]. The 3D SiC_4_ allotropes exhibit a reversible capacity of 176.3 mAh/g and an average open-circuit voltage of 0.55 V, which is safer compared to materials such as 1T-MoS_2_ (1.25 V). They also have a diffusion barrier of 0.41 eV, indicating an acceptable rate capability, comparable to graphite (0.22–0.41 eV), with a minimal volume change of 0.57% during charge/discharge. First-principle simulations of the use of graphitic SiC in NIBs predict that it will offer the highest theoretical capacity for sodium ions (1339.44 mAh/g) among the known NIB anodes, having a low diffusion energy barrier of 0.15 eV [126]. This characteristic enables faster charging and discharging rates and provides moderate open-circuit voltage values during intercalation processes, which could effectively prevent the formation of sodium dendrites. Furthermore, doping with an atom that has a lower electronegativity than carbon enhances the intercalation strength of sodium.

**Figure 7:**
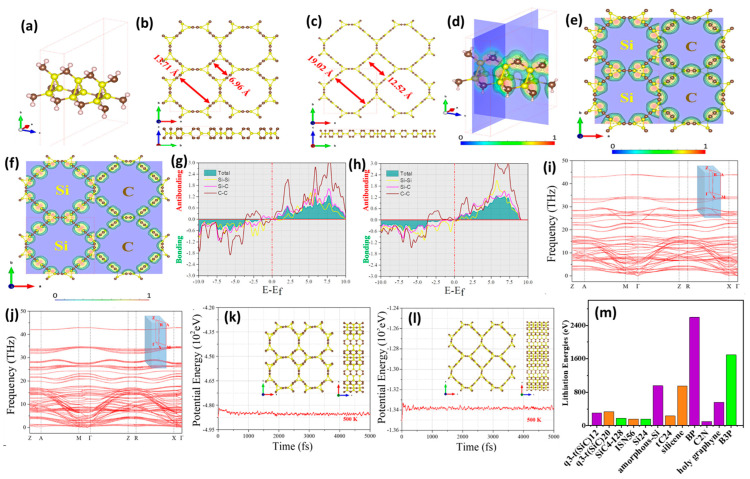
(**a**–**f**) the structural arrangement of quasi-three-dimensional tetragonal SiC polymorphs. (**g**–**l**) Analyzes the stability of the quasi-three-dimensional tetragonal silicon carbon (SiC) polymorphs. (**m**) Migration energy barrier of q3-t(SiC)_12_ and q3-t(SiC)_20_ with other anode materials for Na-ion batteries. Adapted with permission from Ref. [127]. 2023, *ACS Appl. Energy Mater*.

Quasi-3D porous tetragonal silicon carbon polymorphs t(SiC)_12_ and t (SiC)_20_ were investigated using DFT calculations, where both monolayers exhibit robust electronic and band structures characterized by Dirac cones, as demonstrated in Figure 7a–f, positioning them as potential anode materials for NIBs [127]. The energy stability was confirmed through cohesive energy calculations, which indicate bonding strength in the monolayer tSiC, where Si-Si < silicene and C-C > graphene, resulting in greater stability for tSiC. Crystal orbital Hamilton population (COHP) analysis reveals stable chemical bonding in q3-t(SiC)_12_ and q3-t(SiC)_20_ (Figure 7g–l), where all bonds below the Fermi level are stable, except for the slightly occupied C(sp^2^)–C(sp^2^) bond in the antibonding state, attributable to the low electronegativity of Si, which is particularly evident in the porosity of q3-t(SiC)_20_. The absence of soft modes suggests that the polymorphs are structurally resilient to vibrations. Both structures maintained their integrity and demonstrated thermal stability during simulations conducted at 500 K for 5 picoseconds.

The electronic band structures of q3-t(SiC)_12_ reveal a small indirect band gap of approximately 0.59 eV, categorizing it as a semiconductor. In contrast, q3-t(SiC)_20_ exhibits semimetal states characterized by Dirac nodal lines, indicating a high level of conductivity. PDOS analysis indicates that the metallic states in q3-t(SiC)_20_ arise from the aromatic Si and C atomic orbitals. Both q3-t(SiC)_12_ and q3-t(SiC)_20_ demonstrate high theoretical-specific capacities exceeding 300 mAh/g. Additionally, q3-t(SiC)_12_ experiences a volume expansion of 3.9%, which is less than the 5.3% observed in q3-t(SiC)_20_, and both materials exhibit minimal volume changes of less than 6% during Na ion insertion. This performance is superior to that of conventional anode materials such as SiC_4_, ISN, and tC_24_, ensuring mechanical stability. The most stable Na binding site for q3-t(SiC)_12_ has a binding energy of −0.68 eV; this is lower than the binding site for q3-t(SiC)_20_, which is −0.93 eV. At this site, Na-ion transfers are around 0.87 eV, accompanied by superior electrochemical kinetics, as the migration energy barrier for Na-ion diffusion is 0.13 eV for both materials. Na ions migrate effectively through the one-dimensional channels of both polymorphs, as illustrated in Figure 7m, suggesting favorable rate capabilities during charge and discharge cycles. Importantly, the average voltage during the charge and discharge process is suitable for safe NIB operation, minimizing the risk of sodium dendrite formation.

### 3.2. Experimental Studies of Doped SiC Anodes for LIBs

Doping SiC with either heteroatoms (such as N, B, P, O, and halides) or metal atoms can enhance the performance of SiC and Si/C anodes used in metal-ion batteries. This doping process promotes electronic conductivity, enhances the diffusion of metal ions (including Li, Na, and Zn), improves structural stability, and provides active sites for the insertion and extraction kinetics of metal ions. Additionally, it increases wettability, facilitates pseudocapacitive behavior, boosts capacity and rate performance, and improves the uniformity and stability of the solid–electrolyte interphase (SEI) layer. N-doped carbon-coated silicon (N-doped Si/C) materials, created by ball milling Si nanoparticles with asphalt as the carbon source and dopamine as the nitrogen source, followed by annealing, exhibited better cycling stability and a higher initial charging capacity of 2265.1 mAh/g compared to undoped Si/C at 200 mA/g, resulting in a 36.7% capacity enhancement [128]. This improvement is attributed to the N-doped carbon layer, which facilitates Li-ion diffusion and enhances electrical conductivity.

**Figure 8:**
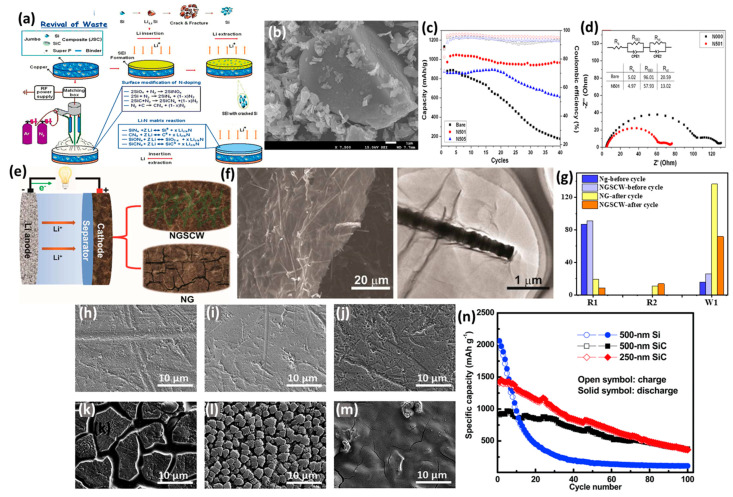
(**a**) Schematic plot for the difference in the amount of waste with/without N-APPJ (recycling waste). (**b**) SEM image of refined waste powder. (**c**) Cycling test and coulombic efficiency of N000, N501, and N505. (**d**) Nyquist plots after the first charging process involving a fitted electronic circuit. Adapted with permission from Ref. [129] 2015, *ACS Appl. Mater. Interfaces*. (**e**) Schematic of the cell configuration and the corresponding electrode structure of NGSCW and NG after cycling. (**f**) SEM, TEM. (**g**) Impedance parameters derived using the equivalent circuit model. Adapted with permission from Ref. [130] 2017, *J. Mater. Chem. A*. SEM images comparing the surface morphology of Si, thin SiC, and thick SiC films, respectively, (**h**–**j**) before and (**k**–**m**) after cycling. (**n**) Cycling characteristics of Si, thin SiC, and thick SiC films at a 0.3 C charge/discharge current. Adapted with permission from Ref. [131] 2018, *RSC Adv*.

A jumbo Si/SiC composite (JSC) of cubic Si particles and hexagonal SiC micro-particles was used as an anode for use in LIBs, which was purified and prepared from the solar power industry waste [129]. The composite (JSC) underwent surface treatment using a N_2_ atmospheric pressure plasma jet (N-APPJ), which introduced N doping (Figure 8a). The purified powder exhibited an uneven morphology, consisting of a JSC with Si particles measuring several hundred nm and SiC particles ~10 μm (Figure 8b). The distribution of Si and N on the electrode surface confirmed that nitrogen doping occurs primarily on both Si and SiC, demonstrating the effectiveness of the N-APPJ-induced surface treatment in modifying the electrodes. The morphological changes in the electrodes after N-APPJ treatment indicate an improvement in structural integrity. The treated electrode showed a smoother and more uniform surface morphology with reduced cracks. The plasma treatment resulted in the formation of a nitride layer that suppressed solid–electrolyte interphase (SEI) formation and minimized volume expansion, compared with the untreated electrode (N000), which displayed excessive cracks and uneven surface morphology due to significant volume expansion during cycling; this lead to a notable increase in thickness and structural instability when compared to the treated electrode (N501). The treated electrodes (N501 and N505) exhibited significant improvements in cycling stability and Coulombic efficiency compared to the untreated electrode (N000). The untreated electrode experienced capacity decay due to severe volume expansion and SEI formation during cycling. Notably, N501 demonstrated a better balance between conductive and insulating compounds compared to N505 and high Coulombic efficiency (Figure 8c), as light dotted lines distinguish efficiency data from solid capacity curves. Consequently, the treated electrode exhibited smoother alloying/dealloying reactions and maintained stable capacity over 40 cycles, unlike the untreated electrode. At the first cycle, the reversible capacity values for the N000, N501, and N505 electrodes were 86.96%, 81.25%, and 71.53%, respectively. The lower value corresponds to greater consumption of Li ions for the L–N and L–O compounds during charge/discharge. The significant performance of the N-APPJ-treated electrodes in promoting the charge transfer ability of Li ions is illustrated in the EIS Nyquist plots, which show a decrease in resistance values for both the solid–electrolyte interphase (SEI), from 96.01 to 57.93 Ω, and charge transfer, from 20.59 to 13.02 Ω for N501 compared to N000. This indicates that a Li–N matrix effectively suppresses and enhances the charge transfer ability of lithium ions (Figure 8d).

A scaffold of SiC whiskers embedded in N-doped graphene (NG) sheets (NGSCW) was prepared via a one-pot thermal nitridation reduction of graphene oxide/SiC foam in an NH_3_ atmosphere, to be used as a cathode for LSIBs. As illustrated in Figure 8e, cross-linked SiC whiskers have been incorporated between the NG sheets [130]. TEM images confirm that SiC whiskers are closely attached to the graphene sheets, exhibiting interplanar distances of 2.52 Å and 2.18 Å in the SiC structure. The NGSCW electrode demonstrates an enhanced capability to maintain a dense and crack-free surface, even after 150 charge/discharge cycles, due to the reinforcing effect of the SiC whiskers. In contrast, the NG electrode, which lacks SiC, develops significant cracks approximately 10 μm wide (Figure 8f). The robust composite structure of the NGSCW is advantageous for LSIB applications, showing cycling stability and coulombic efficiency, with a stable capacity of 1034 mAh/g and a capacity retention exceeding 80%. The lower polarization of NGSCW (170 mV) can be attributed to its superior cathode integrity compared to the NG electrode, which exhibits a lower reversible capacity and reduced stability, reflected by a higher polarization value of 250 mV. This combination effectively accommodates volume expansion and ensures efficient electrical contact between the S reagent and the graphene framework, allowing for consistent electrochemical performance over extended cycles [130]. The effectiveness of the electrochemical reaction was analyzed by the EIS of both electrodes before cycling. A depressed semicircle was displayed in the high-frequency region and a short-inclined line in the low-frequency region, corresponding to patterns of interphase contact resistance and ion diffusion within the cathode. After 150 cycles, notable differences were observed: the NG electrode exhibited higher interphase contact resistance (19.56 Ω) and Warburg resistance (136.4 Ω), indicative of structural degradation. Meanwhile, the NGSCW electrode maintained lower resistances (8.8 Ω and 71.7 Ω, respectively), signifying superior structural integrity and efficient ion/electron transport (Figure 8g). The excellent electrochemical performance of the as-prepared NGSCW cathode, at a current density of 1675 mA/g after 500 cycles, with a capacity retention of over 71%, can be attributed to the mitigation of electrode destruction.

Graphitized SiC (EG/SiC) is prepared through graphitization in an ultrahigh vacuum, resulting in enhanced lithiation capacity and electrical conductivity in LIBs, with a notably high capacity of 34 mAh cm^−2^ and low resistance of 0.2 Ω/square [132]. In contrast, as-received SiC (6H-SiC) shows a low lithiation capacity of 0.6 mAh cm^−2^ and sheet resistance exceeding 10 MΩ/square. This improvement is attributed to the critical roles of doping and thermal treatment, which help remove the native oxide layer and introduce surface defects. The nc-SiC film anode, fabricated using modified plasma-enhanced chemical vapor deposition (PECVD), enhances LIB performance through improved capacity retention and cyclability. The thinner films (150 nm) exhibit a reduced capacity fade of less than 21%, with a capacity of 309 mAh/g, compared to the 600 nm thick films, which show a capacity of 230 mAh/g and no observable cracks after 60 cycles, due to the formation of a Li_4_C buffer matrix and their structural characteristics [133]. The MAX phase (Ti_3_SiC_2_), prepared by anodization for use as an anode material in Li-ion microbatteries, demonstrates a relatively stable capacity, with an applied current density of 500 μAh·cm^−2^ and a coulombic efficiency exceeding 95% over 60 cycles [134]. It maintains stable areal capacities of 380 μAh·cm^−2^ after 140 cycles, benefiting from enhanced kinetics due to its porous structure and the presence of anatase, silica, and carbon, in contrast to pristine Ti_3_SiC_2_, which shows no significant electrochemical activity. Anodized Ti_3_SiC_2_, used in Li-ion microbatteries, exhibits a nanolayered structure at 10 V that provides superior pseudocapacitive charge storage and a higher power density of 38 μW cm^−2^ μm^−1^ due to its increased porosity and larger surface area [135]. In comparison, the mesoporous morphology at 60 V yields a power density of 14 μW cm^−2^ μm^−1^, relying more on bulk diffusion for charge storage. Hollow mesoporous MnO/MnS/SiC/S-CN-x (where x represents MnO_2_ contents) anodes were synthesized using a hydrothermal-assisted method with soda pulp black liquor as a template; these were subsequently employed for use in LIBs [136]. The MnO/MnS/SiC/S-CN-2 composites outperform other variants, including MnO/MnS/SiC/S-CN, MnO/MnS/SiC/S-CN-1, and MnO/MnS/SiC/S-CN-3, maintaining a 100% stable charge/discharge capacity of 837 mAh/g and 756 mAh/g at a current density of 1 A/g after 50 cycles. This performance exceeds the theoretical capacities of MnO (755 mAh/g) and MnS (616 mAh/g), attributed to the synergistic effects between MnS and MnO, as well as the optimized hollow mesoporous structure.

The amorphous SiC film (500 nm) was prepared using inductively coupled plasma chemical vapor deposition (ICP-CVD) at a relatively low processing temperature of 350 °C, utilizing Ar, SiH_4_, and CH_4_ as precursors [131]. This low temperature facilitates the formation of an amorphous structure, which enhances the anode’s performance. Three film thicknesses were prepared: pure Si (500 nm), SiC (250 nm), and SiC (500 nm) (Figure 8h–j). The thicker SiC film, after cycling, exhibited a few hairline cracks but demonstrated superior mechanical stability (Figure 8k–m) and was associated with better capacity retention compared to the other anode films. In contrast, the Si and thin SiC anodes fractured due to significant volume changes induced by repeated cycling, resulting in poor capacity retention. The SiC sample exhibited similar voltage profiles to the Si sample in subsequent cycles, suggesting comparable electrochemical reactions occurring between them. The initial charge/discharge capacities of the Si and SiC samples were 3787 mAh/g/4110 mAh/g and 1371 mAh/g/1595 mAh/g, respectively, corresponding to coulombic efficiencies (CE) of 92.1% and 86.0%. The SiC sample demonstrated a lower initial capacity and CE than the Si sample, likely due to incomplete reactions occurring in the SiC anode. The enhanced electrochemical performance of the thick SiC film was indicated by an initial reversible specific capacity of 917 mAh/g, with a capacity retention of 41.0% after 100 cycles. Although the thin SiC film exhibited a higher initial capacity (1427 mAh/g), it had poorer retention (25.7%). In comparison, the pure silicon (Si) anode film showed significantly poorer retention (5.2%) (Figure 8n). The better capacity retention of the thick SiC sample relative to the thin one suggests that residual SiC plays a role in capacity retention. In the thick film, SiC serves as a buffering matrix, mitigating volume-induced stress and protecting the active material from mechanical degradation. Consequently, the hardness of the film would decrease rapidly following Li^+^ insertion.

Porous Si/SiC composite (pSi/SiC) spheres prepared through magnesiothermic reduction serve as a suitable anode material for LIBs [137]. They demonstrate exceptional specific capacities of 1653.4 mAh/g and 1446.7 mAh/g at 0.5 A/g and 1 A/g after 100 cycles, 1022 mAh/g at 2 A/g after 400 cycles, and 420 mAh/g at 5 A/g even after 2000 cycles. This performance emphasizes their superior Li-storage capability in comparison to porous carbon-coated Si, Si/rGO films, porous Si/C, and other Si-based anode materials. These materials exhibit reduced charge transfer resistance relative to bare pSi, attributable to the maintenance of the structural integrity of the porous silicon, effective buffering of volume expansion, and optimized pore distribution. Furthermore, 3D frameworks of SiC and Si@CNTs, created through an in situ reduction method, have shown promise as potential anode material for LIBs. The most effective composites, SiC and Si@CNTs-1, achieved a high specific capacity of 1051.44 mAh/g after 880 cycles at 1 A/g, along with superior rate capability and stable cycle performance [120]. This is due to SiC’s role in reducing electrode degradation and stabilizing the solid–electrolyte interphase (SEI) layer, especially when compared to the significant degradation observed in Si@CNTs, which dropped to 202 mAh/g in capacity and exhibited 200% volume expansion over 100 cycles. Carbon/SiC/SiNPs composites reveal their potential for enhancing LIB anodes, leading to improved electrochemical performance [138]. This includes higher capacity retention, better cycling stability, and enhanced rate capability. Advanced designs, such as yolk–shell structures, further optimize these characteristics by accommodating volume changes, minimizing side reactions, and providing high durability and fracture toughness. In contrast, SiNP-based materials are more susceptible to thermal degradation and require structural modifications for improved stability. Nanoporous Si, macro-honeycomb porous Si, and SiC nanocomposites have been synthesized via magnesiothermic methods to function as professional anodes in LIBs [139]. SiC demonstrates superior long-term performance and cycling stability, initially discharging to 1125 mAh/g, which is 78% of the theoretical capacity, and achieving 85.7% charge retention after the second cycle following an aging period. In comparison, Si-nano reaches only 700 mAh/g with 40% irreversibility and 47.8% charge retention. This difference is primarily due to SiC’s ability to effectively manage strain and volume changes, attributed to interstitial carbon atoms within its structure. Additionally, the improved kinetics results in the lowest charge transfer resistance for SiC at 36.5 Ω, compared to 152 Ω for Si-nano and 217 Ω for Si-macro, highlight the stability of the interface and the SEI layer. Porous Si, protected by a SiC/C dual interface prepared through a conventional exothermic displacement reaction, exhibits an enhanced initial Coulomb efficiency of 75.0%, compared to a previous efficiency of 72.3% [140]. It also shows superior cycle stability at 731.1 mAh/g versus 494.0 mAh/g after 200 cycles at 0.4 A/g, and improved rate performance at 903.1 mAh/g compared to 375.5 mAh/g at 3 A/g, relative to the reference. The porous Si/C sample benefits from the protective effects of porous Si/SiC/C, which acts as a buffer against mechanical stress during electrochemical cycles. Hierarchical carbon-coated Si nanofiber Si/C, prepared via a two-step chemical vapor deposition method and one-step in situ polymerization, serves as an anode for LIBs and displays excellent rate capability with a capacity retention rate of 89.8% under varying currents [141]. It retained a capacity of 528.3 mAh/g after 100 cycles at 100 mA/g, showing no noticeable structural damage and suppressed Si volume expansion. This suppression is attributed to strengthened bonding between silicon and CNF due to the formation of SiC at the interface. Porous Ti_3_SiC_2_-derived carbon (Ti_3_SiC_2_–CDC), produced by conventional etching methods, also functions as an anode for LIBs and exhibits remarkable cycling stability and reversible capacity [142]. This material outperformed CDCs prepared at higher temperatures and competing materials, achieving a reversible capacity of 712.9 mAh/g after 300 cycles. It maintained good performance at higher current densities (100 mAh/g) and demonstrated a highly reversible capacity of 375.6 mAh/g, even at a current density of 500 mAh/g, showcasing excellent rate capability. The C@SiC@Si@SiC@C structure, developed through a polymer-directed strategy, acts as a performance anode in LIBs that shows a high utilization of the active substance [143]. It achieves an outstanding measured capacity of 3200 mAh/g with an ultra-low decay rate of 0.7‰ per cycle, while maintaining nearly 100% Coulombic efficiency at 0.2 C. Such superior performance is linked to its uniquely designed structure. The C/SiC composite produced via carbothermal synthesis serves as anode half-cells for LIBs, demonstrating superior discharge capacities of 328 mAh/g and 400 mAh/g at a current of C/2 for mixtures of (wt%) 29.5C–70.5SiC and 50Si–14.5C–35.5SiC, respectively. In contrast, pure SiC reached discharge capacities of only 180 mAh/g and 138 mAh/g at current rates of C/20 and C after 100 cycles, while maintaining over 99% Coulombic efficiency during cycling [144].

**Figure 9:**
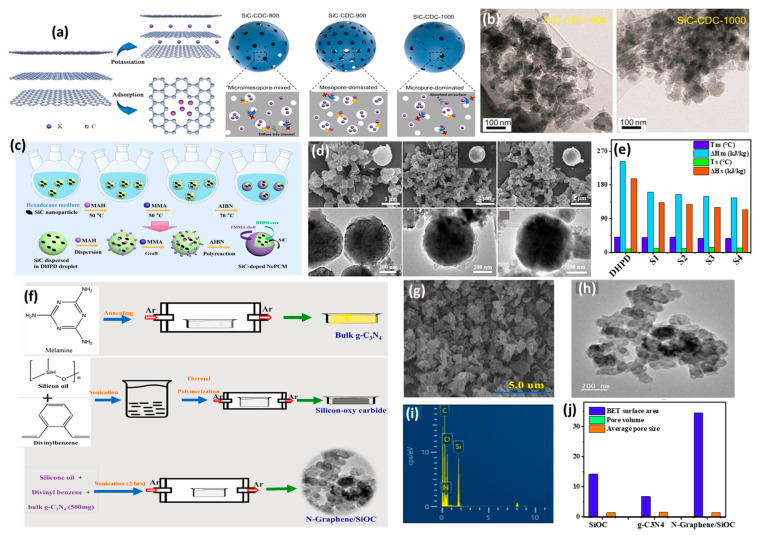
(**a**) Schematic plots of the mixed mechanisms of K+ storage and diffusion processes of K+ ions inside the SiC-CDC anodes. (**b**) TEM images of SiC-CDC-900 and SiC-CDC-1000 anodes. Adapted with permission from Ref. [145] 2020, Adv. Funct. Mater. (**c**) Schematic of the synthesis process and mechanism of SiC-doped NePCMs, (**d**) SEM and TEM images of SiC-doped NePCMs. (**e**) Phase-change properties of DHPD and NePCM with different ratios of SiC. Adapted with permission from Ref. [146] 2022, *ACS Appl. Energy Mater*. (**f**) Schematic illustration of the synthesis of bulk g-C_3_N_4_, silicon-oxy carbide, and N-graphene/SiOC. (**g**) SEM (**h**) TEM (**i**) EDX mapping of N-graphene/SiOC. (**j**) The BET surface area, pore volume, and average pore size of g-C_3_N_4_, SiOC, and N-graphene/SiOC. Adapted with permission from Ref. [147] 2023, *J. Energy Storage*.

The structure of silicon-carbide-derived carbon (SiC-CDC), which consists of abundant interconnected pores, primarily mesopores and micropores, was prepared by thermally etching SiC at various temperatures (800 °C, 900 °C, and 1000 °C), resulting in differing pore distributions and surface areas that work as anodes for use in KIBs (Figure 9a) [145]. The aerogel-like framework of SiC-CDC-900, which features interlinked spherical particles with vesicular textures, shows less-homogeneous porous regions and shorter-range stacked graphitic layers compared to SiC-CDC-800. In contrast, SiC-CDC-1000 presents distinct graphitic fringes (0.335 nm) alongside amorphous carbon (Figure 9b). The materials exhibit a combination of micropores and mesopores; moreover, the SiC-CDC-900 anode is notable for having the highest specific surface area (SSA) of 2205 m^2^/g, the largest mesopore volume of 1.07 cm^3^/g, and an optimal pore size distribution. Its excellent reversibility is demonstrated by retaining 192.0 mAh/g even after 1000 cycles at a current density of 1.0 A/g, resulting in 89.3% capacity retention. Additionally, its Coulombic efficiency approaches 100% when compared to SiC-CDC-800 and SiC-CDC-1000, underscoring its durability. The superior performance of SiC-CDC-900 in terms of ion transport and reaction kinetics is evidenced by its lower charge transfer resistance (Rct) relative to SiC-CDC-800 and SiC-CDC-1000. Consequently, the high performance of SiC-CDC-900 can be attributed to its mesopore-dominated structure, which facilitates a high ratio of capacitive contribution, enhanced ion kinetics, and efficient potassium storage. This structure enables better ion diffusion and more dynamic reaction kinetics compared to micropore-dominated anodes like SiC-CDC-1000. SiC-doped nanoencapsulated phase-change materials (NePCMs) are engineered for battery thermal management systems in LIBs and created by uniformly dispersing hydrophilic SiC nanoparticles in a hydrated salt solution. Using ultrasound, high-speed shearing results in a stable water-in-oil emulsion. Methyl methacrylate (MMA) is then introduced to the emulsion, where it polymerizes under heat and in the presence of a catalyst, forming a polymethyl methacrylate (PMMA) shell around the hydrated salt and SiC core (Figure 9c) [146]. The SiC NPs are fully encapsulated within a spherical structure with smooth surfaces and an average diameter of 400–600 nm, while individual nanoparticle sizes range from 10 to 60 nm (Figure 9d). The thermal stability of the phase-change enthalpy of the NePCMs remains excellent, showing only a 2.7% decrease after 400 heating–cooling cycles, compared to a loss of over 60% enthalpy after just 10 cycles for pure hydrated salt. The thermal conductivity of NePCMs, both in the solidified state (25 °C) and melted state (50 °C), is enhanced by the presence of SiC nanoparticles compared to NePCMs without SiC. This enhancement is due to the high thermal conductivity of SiC nanoparticles (179–393 W/m·K) and their ability to create a thermally conductive network within the capsules. The superior corrosion resistance of SiC-doped NePCMs is evident, as the PMMA shell effectively protects them when exposed to Fe plates. This is in contrast to pure hydrated salt, which showed significant rust and discoloration after 24 h at 60 °C. Furthermore, the NePCM plate exhibited outstanding fire resistance when exposed to a flame of 500–600 °C for 120 s, showing no signs of ignition or disintegration. The effectiveness of NePCMs in temperature regulation, particularly in preventing thermal runaway, is demonstrated by batteries cooled with NePCMs, which maintained a safe surface temperature of around 36 °C during a 2C charging rate, compared to over 60 °C with natural air cooling (Figure 9e).

Thin-film silicon-air batteries utilize n-type amorphous silicon (a-Si) and amorphous silicon carbide (a-SiC), which are prepared through plasma-enhanced chemical vapor deposition (PECVD), serving as anodes for metal-air batteries [148]. These batteries potentially exhibit a high theoretical specific energy of 8470 Wh/kg, which is superior to other metal-air batteries such as aluminum-air (8100 Wh/kg) and zinc-air (1370 Wh/kg). Current commercial zinc-air batteries achieve specific energies of 350–470 Wh/kg, using simpler and safer low-concentration alkaline solutions like KOH or NaOH. Inert model single-crystal nitrogen-doped silicon carbide (N–SiC) electrodes, studied through a multimodal experimental and theoretical approach, serve as a model system to investigate the formation and growth mechanisms of LiF in the solid–electrolyte interphase (SEI) [149]. N–SiC allows for differentiation between two proposed mechanisms of LiF formation: direct anion reduction, and the electrocatalytic transformation of HF. This study indicates that LiF nucleates through HF transformation and subsequently grows via anion reduction. Additionally, porous silicon carbon/nitrogen-doped graphene (Si-C/NG) prepared through a calcination process and magnesiothermic reduction serves as an anode material in LIBs, demonstrating significant electrochemical characteristics, including cycling stability and rate performance [150]. Notably, the Si-C/NG-Mg_0.3_ electrodes exhibited the highest specific capacity, approximately 500 mAh/g at 0.1 A/g after 30 cycles; these outperformed the Si-C/NG-Mg_0.5_ and Si-C/NG-Mg_0.7_ electrodes, which contained lesser amounts of SiC and thus had poorer conductivity, and surpassed the performance of commercial graphite.

Nitrogen-doped graphene/silicon-oxycarbide (N-graphene/SiOC) nanosphere composites were prepared as materials for LIBs following the synthesis of SiOC, which involved incorporating bulk g-C_3_N_4_ into the precursor mixture [147]. At a temperature of 900 °C, bulk g-C_3_N_4_ undergoes thermal reduction to form a nitrogen-doped graphene phase that integrates with the SiOC structure (Figure 9f). The N-graphene/SiOC composite exhibits an amorphous character (Figure 9g) and an agglomerated spherical morphology (Figure 9h), featuring a uniform distribution of elements. The atomic percentage analysis reveals a lower silicon content of 7.69%, while carbon (C) is at 73.86%, nitrogen (N) is at 1.97%, and oxygen (O) is at 16.49% in the N-graphene/SiOC composite (Figure 9i). N-graphene/SiOC possesses a significantly higher surface area of 34.42 m^2^/g compared to SiOC at 14.76 m^2^/g and g-C_3_N_4_ at 6.76 m^2^/g. This enhancement is attributed to the transformation of g-C_3_N_4_ into N-graphene, which increases the number of active sites available for electrolyte–ion interaction. The as-made materials were used as anodes for LIBS, and varied in their performance. The initial charge and discharge capacities for N-graphene/SiOC are superior to those of g-C_3_N_4_ and SiOC, measuring 925 mAh/g and 1324 mAh/g at a current density of 100 mAg^−1^, respectively, with coulombic efficiency stabilizing at around 100% in subsequent cycles. The incorporation of nitrogen-doped graphene (N-graphene) into SiOC substantially increases the surface area, more than doubling that of pure SiOC, while maintaining a similar pore volume and size. This indicates enhanced accessibility and storage potential for Li^+^ ions (Figure 9j). The Nyquist plots indicate charge transfer resistance (Rct) values for g-C_3_N_4_, SiOC, and N-graphene/SiOC at 132.62 Ω, 261.13 Ω, and 149.21 Ω, respectively. The Warburg factor (σ), which reflects lithium-ion diffusion efficiency, is lowest for N-graphene/SiOC at 1.7, suggesting improved ion transport due to the structural characteristics of the composite. This study indicates the significant effect of N-graphene on promoting the performance of SiOC in LIBs. Recently, N-doped graphene (NG) on SiC (NG@SiC) served as an intrinsic electric field with intensive interfacial interaction via electron/ion bridges for stable and efficient LIBs [151]. Both experimental and DFT simulations have demonstrated that the NG@SiC anode demonstrates a reversible capacity (1197.5 mAh g^−1^ after 200 cycles at 0.1 A/g), with a capacity retention of 76.6% after 1000 cycles. Also, the LIB cell containing (LiFePO_4_/C//NG@SiC) displays a superior rate capability and long life cycle due to the NG effect, which enhances the interfacial interaction tailoring strategy.

**Figure 10:**
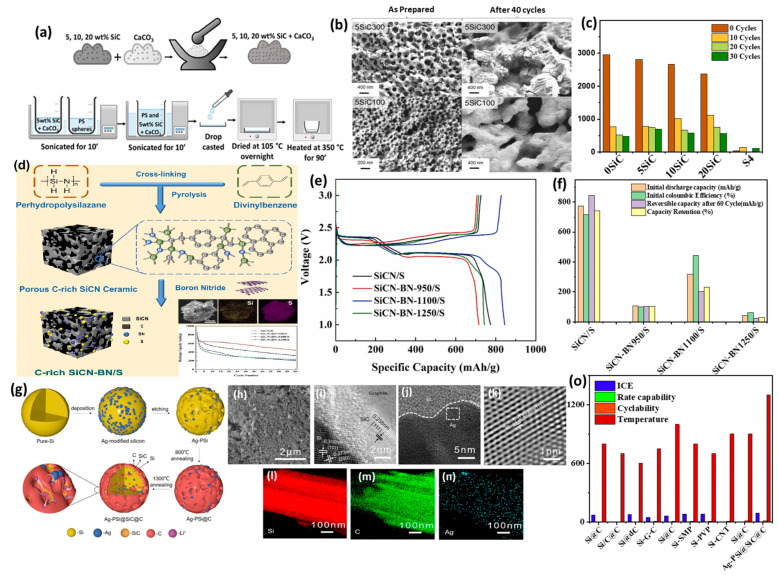
(**a**) The preparation methods for SiC/CaCO_3_ samples. (**b**) The morphological changes in the porous SiC/CaCO_3_ samples (5SiC300 and 5SiC100) before and after 40 calcination–carbonation cycles. (**c**) Heat storage density in kJ/kg for pure CaCO_3_, bulk, and porous SiC/CaCO_3_ samples as a function of the number of cycles. Adapted with permission from Ref. [156] 2023, *J. Alloys Compd*. (**d**) Schematic and SEM images showing cycling performance of the SiCN/S and SiCN-BN/S composites. (**e**) Lithiation curves and delithiation curves of SiCN/S and all SiCN-BN/S samples. (**f**) Electrochemical parameters of the sulfurized SiCN samples. Adapted with permission from Ref. [158] 2024, *J. Alloys Compd*. (**g**) Schematic representation of the fabrication processes for Ag-PSi, Ag-PSi@C, and Ag-PSi@SiC@C, as well as the insertion and extraction of Li^+^ (enlarged diagram). (**h**) SEM image of (Ag-PSi@SiC@C. (**i**–**k**) HR-TEM images of Ag-PSi@SiC@C. (**l**–**n**) EDS element mapping images of Ag-PSi@SiC@C. (**o**) Comparison of the electrochemical performance of the Si-based anode. Adapted with permission from Ref. [159] 2024, *J. Colloid Interface Sci*.

Zinc-blende-type silicon carbide (β-SiC) treated with hydrofluoric acid (HF) serves as an anodic material for LIBs, with a specific capacity of approximately 930 mAh/g; this was significantly higher than the ~79 mAh/g capacity of pristine SiC. Treated β-SiC retains about 80% of its capacity after 175 charge–discharge cycles when paired with a LiCoO_2_ cathode [152]. PVDF-SiC@Zn was synthesized using the spin-coating method for use as an anode in ZIBs. This anode demonstrates stable performance over 780 h due to its low nucleation overpotential and interfacial impedance. It retains 68% capacity after 400 cycles at a current density of 0.1 A/g. The homogeneous Zn deposition and inhibition of Zn dendrite growth make it superior to the bare Zn anode, which experiences erratic voltage fluctuations and rapid capacity decay due to dendrite growth. The modified anode maintains a high coulombic efficiency of 99.3% over 100 cycles, demonstrating excellent reversibility and stability [153]. Porous SiC powders synthesized via the magnesiothermic reduction method serve as anodes in LIBs. They exhibit an impressive specific power and energy density of 3000 W/kg at 1.25 A/g after 130 cycles. In comparison, a silicon anode stabilizes at around 400 W/kg at higher current rates, showing stable reversibility. Coulombic efficiency improves from 91% in the first cycle to 99%, making it preferable to a conventional silicon anode [154]. It also shows a low ohmic resistance of 24.5 after extended use, enhancing charge transfer and cycling efficiency. SiC decorated with nickel nanoparticles (Ni/SiC), prepared by ball milling, is used as thermal filler within an aramid nanofiber (ANF)-based composite film. This composite offers a high thermal conductivity of 0.502 W m^−1^ K^−1^, which is 298.4% higher than that of the original ANF film (0.126 W m^−1^ K^−1^). Additionally, the decomposition temperature improves from 490 °C for pure ANF to 525 °C with 30 wt% filler, indicating the enhanced thermal stability and puncture resistance of the ANF composite [155]. A porous SiC/CaCO_3_ composite is utilized as a high-temperature thermal battery material for thermochemical energy storage in concentrated solar power (CSP) systems. It is prepared by heating SiC and CaCO_3_ in varying weight percentages (5%, 10%, and 20% SiC) to remove moisture before physical mixing [156]. This mixture incorporates polystyrene (PS) spheres (300 nm and 100 nm) as a sacrificial template. The spheres undergo thermal treatment to produce porous SiC/CaCO_3_ (Figure 10a). The resulting structure exhibits well-defined grains of SiC and significantly fewer sintered CaCO_3_ grains after cycling. The specific surface area before and after cycling is measured at 0.28 ± 0.003 and 4.67 ± 0.05 m^2^/g, respectively (Figure 10b). Figure 10c indicates that while a higher SiC content improves initial performance, long-term cycling stability benefits from lower SiC concentrations. All SiC/CaCO_3_ mixtures display superior structures that facilitate faster reaction kinetics compared to pure CaCO_3_. Notably, the 20SiC sample achieves near-complete calcination/carbonation at 884 °C within 20 min during initial cycles, attributed to the high thermal conductivity of SiC, which enhances heat diffusivity. Among the samples, 5SiC300 (larger pores) outperforms others in the first 10 cycles, achieving an effective conversion of 0.29. However, its performance declines significantly after 20 cycles due to pore collapse. Conversely, 5SiC100 (smaller pores) follows a similar trend but experiences a faster decline, reaching an effective conversion of 0.09 by the 40th cycle. The 20SiC sample achieves near-complete calcination/carbonation at 884 °C within 20 min during the initial cycles, which is attributed to SiC’s high thermal conductivity enhancing heat diffusivity. In the first 10 cycles, 5SiC300 (with larger pores) outperforms other samples, achieving an effective conversion of 0.29; however, its performance declines significantly after 20 cycles due to pore collapse. Meanwhile, 5SiC100 (with smaller pores) follows a similar trend but experiences a faster decline, reaching an effective conversion of 0.09 by the 40th cycle. 5SiC300 (300 nm pores) exhibits faster carbonation kinetics compared to 5SiC100 (100 nm pores) due to its larger pore sizes, which allow CO_2_ gas to travel more efficiently through the porous network. Additionally, its performance deteriorates after 20 cycles as the porous structure collapses, resembling bulk samples. As a result, the specific surface area increases significantly for both samples, with 5SiC300 reaching 5.59 ± 0.06 m^2^/g and 5SiC100 achieving 6.31 ± 0.06 m^2^/g.

C-rich silicon carbonitride (SiCN) prepared by the solvothermal method serves as a cathode for LSBIs [157]. The low-temperature pyrolyzed samples (SiCN-S-1000) which have a higher sulfur content of 66 wt% compared to other cathode materials, demonstrate superior reversible capacity (313 mAh/g). This capacity outperforms that of SiCN-S-1200 (251 mAh/g), SiCN-S-1400 (230 mAh/g), and SiCN-S-1600 (214 mAh/g). Furthermore, SiCN-S-1000 exhibits better capacity retention (43%) than SiCN-S-1200 (27%), SiCN-S-1400 (32%), and SiCN-S-1600 (25%). This improved performance is attributed to its amorphous carbon phase and higher nitrogen content, which help suppress polysulfide migration. Likewise, a composite of polymer-derived silicon carbonitride (SiCN) ceramic matrix combined with BN nanosheets (SiCN-BN/S) serves as a host for S cathodes in LSIBs [158]. The uniformly distributed SiCN-BN/S composites are prepared by dispersing urea, boric acid, and the synthesized SiCN ceramic in deionized water, followed by heating to evaporate the water. The resulting solid is then annealed at varying temperatures (950 °C, 1100 °C, and 1250 °C) under argon to form SiCN-BN composites. These composites undergo ball milling with sulfur in a sealed autoclave for 24 h, followed by grinding. Moreover, the irregular rough surfaces of the SiCN-BN/S/S composites, with uniformly distributed S are confirmed within the given resolution limit, as shown in Figure 10d. All measured samples exhibit mesoporous characteristics, with both specific surface area and total pore volume decreasing as the annealing temperature increases. SiCN-BN-1100 contains pores in a size range of 2 to 180 nm and reveals a significant amount of small mesopores (3 to 10 nm), which are formed due to carbothermal reduction (as indicated by elemental analysis results). SiCN-BN-950/S demonstrates high reversibility with nearly 100% initial coulombic efficiency, indicating the improved reversibility of the sulfur cathode. In contrast, the increased mesopores and graphitization in SiCN-BN-1100/S/S confirm a notably high initial discharge and charge capacity (Figure 10e). SiCN-BN-950/S strikes the best balance between stability and efficiency concerning the impact of annealing temperature on electrochemical properties (Figure 10f). The SiCN-BN-950/S sample retained the highest discharge capacity and capacity retention in all subsequent cycles, achieving a reversible capacity of 445 mAh/g, while SiCN/S showed only 319 mAh/g. The capacity retentions for SiCN-BN-950/S and SiCN/S after 60 cycles were 62% and 41%, respectively. SiCN-BN-950/S also demonstrates the superior rate performance, surpassing other samples at higher cycling currents (e.g., 0.1 C and above).

A sandwich structure of porous silicon (PSi) encapsulated in a bilayer of carbon/silicon carbide/silver-modified porous silicon (Ag-PSi@SiC@C) was developed as an anode for high-performance LIBs. This was achieved by depositing Ag on Si, followed by etching to create PSi, and then coating with glucose. The structure underwent consecutive annealing at 800 °C and 1300 °C to facilitate the formation of SiC (Figure 10g) [159]. Ag-PSi@SiC@C exhibited a uniform porous structure with well-encapsulated Ag nanocrystals (Figure 10h). The bilayer structure was confirmed through TEM, which revealed SiC with a thickness of 5–10 nm, well-attached to Ag-PSi, along with a carbon (C) layer measuring 5–8 nm in thickness, and resolved a d-spacing of Si, SiC, and Ag (Figure 10i–k). This suggests the creation of a homogeneous composite, as further illustrated by EDX mapping, which demonstrated a uniform distribution of Ag, Si, and C in Ag-PSi@SiC@C (Figure 10l–n). Consequently, Ag-PSi@SiC@C enhanced Li-ion diffusion in the anode/cathode regions, with a measured value of 2.4 × 10^−9^ cm^2^/s in the cathode region, outperforming Ag-PSi and Ag-PSi@C. Thus, the initial specific capacity of 3637.5 mAh/g and coulombic efficiencies (CEs) of 90.1% for Ag-PSi@SiC@C were significantly superior to those of pure Si, Ag-PSi, and Ag-PSi@C. Additionally, it maintained a specific capacity of 962.6 mAh/g, and CEs of 98.4% after 200 cycles at a current density of 2 A/g. The superior performance of Ag-PSi@SiC@C stems from the porosity of its sandwich structure, which enhances accessibility to active sites and improves ion diffusion and mobility. This is evidenced by the Nyquist plot, which showed lower charge transfer resistance and the highest solid–electrolyte interphase (SEI) capacitance for Ag-PSi@SiC@C. For practical battery applications, Ag-PSi@SiC@C exhibited a discharge capacity of 142.7 mAh/g at a rate of 0.1 C and retained 98.9 mAh/g after 150 cycles at 1.0 C when paired with a LiFePO_4_ cathode. Compared to various SiC-based electrodes, Ag-PSi@SiC@C demonstrated superior performance and durability (Figure 10n).

## 4. Conclusions and Future Perspectives

This review has outlined the predominant synthetic strategies for fabricating SiC- and Si/C-based composite anodes for rechargeable batteries (i.e., LIBs, NIBs, ZnBs, and PIBs), with particular emphasis on ball milling, pyrolysis, chemical vapor deposition, and spray-drying methods. Each method offers distinct advantages, but pyrolysis stands out as a particularly promising route due to its simplicity and compatibility with other synthesis techniques, which make it suitable for scalable production (Table 1). The incorporation of a conductive carbon layer onto SiC and Si/C composites significantly enhances electrochemical performance by improving electrical conductivity, precluding volume changes, prolonging life cycle, and increasing specific capacity (Table 2). Additionally, heteroatom doping (e.g., N, P, O, B) has shown substantial promise in enhancing electrochemical properties by lowering charge-transfer resistance, boosting electrolyte–electrode interaction, and improving structural stability (Table 2).

Despite these advancements, several critical challenges must be addressed before the practical implementation of SiC- and Si/C-based anodes for metal-ion batteries can be realized at an industrial scale:

- Performance Limitations: The currently reported Li-storage capacities of SiC and Si/C materials (≤1500 mAh/g), as well as cycling stability (≤500 cycles), are still insufficient for commercial utilization. The practical industrial energy storage deployment of Si/C and SiC requires a high energy density, long cycle life, and high charging/discharging rates. Mainly, their capacity should reach tens or hundreds of kilowatt-hours per cycle life (≥3000 cycles). Overcoming these performance limitations may be solved via integrating high-capacity active materials such as porous-activated biochar [160], MXenes [161], and graphdiyne [162]. These materials offer outstanding surface area, electrical conductivity, specific capacity, huge Li-storage capacity, and physiochemical stability in addition to ease of preparation from earth-abundant materials, which are needed merits for the industrial battery devices.
- Synthesis Complexity: The methods of forming of SiC composites are not productive (up to the milligram range) and entail multiple reaction steps and heating to elevated temperatures, leading to improper and inhomogeneous distribution of Si within the skeleton structure of carbon [28,51,52,53]. Thus, these methods should be simplified and result in a higher yield (up to the kilogram range), besides providing a homogeneous coating of Si with carbon to fit scalable and practical usage. These issues could be resolved via the in situ growth of Si nanoparticles within carbon support by means of chemical reduction, seed-mediated, templates, and microwave methods, along with their coupling, to be feasible for commercial utilization.
- Unresolved Electrolyte Effects: The effect of electrolytes on the performance of SiC and Si/C anodes is still ambiguous and not yet resolved. Using organic electrolytes (i.e., ionic liquids and poly (ionic liquid) solid polymer electrolytes) [19,163] could probably widen the potential window and enhance the specific capacity and life cycle of SiC and Si/C anodes. Also, these electrolytes can improve safety and thermal stability, enhance energy density, and preclude Si-volume change or metal-ion dendrite growth, thus offering a pathway toward enhanced long-term cycling performance.
- Underexplored Doping Strategies: The effect of heteroatom doping (i.e., N, B, P, O, and halides) and metal atoms on the performance of SiC and Si/C anodes for metal-ion batteries is rarely reported; meanwhile, understanding distribution, interaction, and stability under cycling conditions remains a key challenge. The integration of these heteroatoms can pave the way for hot research directions and enhance the battery performance [164,165]. This is due to the significant effects of heteroatoms, resulting in improved electronic conductivity, enhanced metal-ion (Li, Na, and Zn) diffusion, structural stability, and the creation of active sites for metal-ion insertion/extraction kinetics [128,151]. In addition to increasing the wettability, providing pseudocapacitive behavior, and boosting capacity and rate performance, these heteroatoms improve SEI layer uniformity and stability.
- Scalability Challenges and Bottlenecks: From the industrial viewpoint, the key scalability hurdles include inconsistent material quality at the large scale, the high energy demand for synthesis, and integration challenges with current industrial fabrication processes for the electrodes of metal-ion batteries. The industrial case studies indicate that translating lab-scale pyrolysis or CVD methods into continuous, roll-to-roll production remains impractical, owing to reactor design limitations, gas management, and precursor control. Solving these barriers will require process optimization, modular reactor designs, and improved precursor delivery systems.
- Necessity of Using Machine Learning and Simulations: Machine learning (ML) offers transformative opportunities across multiple stages of SiC and Si/C anode development and performance predictions. Machine learning models can easily be used for the discovery of SiC and Si/C materials by predicting optimal dopant combinations or structural motifs with a high capacity and low volume expansion. In addition to performance prediction, they correlate synthesis parameters of Si/C and SiC with electrochemical performance. Meanwhile, ML can assist process optimization by fine-tuning preparation method conditions and factors to ensure consistent product quality. Also, ML-guided models can be used to predict optimal electrolyte conditions (i.e., type, composition, and pH) for metal-ion batteries. Coupling ML with density functional theory (DFT) calculations could accelerate the identification of high-performance compositions and interface chemistries, leading to more efficient SiC and Si/C material designs [166,167,168]. This endeavor works to optimize the performance of SiC and Si/C anodes (i.e., shape, composition, and strain/synergetic effect) for various metal rechargeable batteries, especially NIBs, ZnBs, and PIBs, to complement the extensive existing research on LIBs.
- Advanced Characterization Needs: In situ characterization techniques such as SEM, TEM, and XPS should be further integrated and synchronized during battery cycling to elucidate structural evolution, SEI layer formation, and degradation pathways [169]. Such insights will be crucial for rational SiC and Si/C anode design and lifespan extension.

## Figures and Tables

**Table 1:** Comparison table between various methods used in the preparation of SiC.

Method	Yield/Purity	Cost	Scalability	Particle Size	Method Performance	Battery-Relevant Performance
Pyrolysis	High/Moderate	Low	High	Controlled (10–100 nm)	Moderate	- Good control of Si/C ratio. - Improves cycle life. - Porous structure with moderate surface area.
Ball Milling	Moderate/Low	Moderate	High	Non-controlled (Broad agglomerated NPs)	Low to Moderate	- Poor crystallinity, low conductivity, surface defects, and contamination. - Poor electrochemical stability. - Requires post-treatment.
Spray Drying	Moderate/Moderate	High	Low	Controlled (depend on nozzle and drying control)	Moderate	- Good powder flowability. - Useful for structured electrodes. - Medium conductivity.
Chemical Vapor Deposition (CVD)	Very Low/High	Very High	Low	Excellent control (<50 nm films or shells)	High (crystalline, phase-pure)	- High conductivity. - Excellent structural stability as well as cycling and mechanical integrity.

**Table 2:** Comparison of SiC and Si/C composite anodes for metal-ion batteries.

Composite	Modification	Advantages	Disadvantages	Electrochemical Performance	Ref.
C@void/Si-g	Carbon coating via pitch + NaCl template	Improved durability; Si isolation from electrolyte	Template removal; multistep	LIB: 1082.7 mAh/g after 200 cycles at 0.2 C	[60]
Si30@C40/G30	Graphene + sucrose carbon coating	High capacity and cycle stability	High irreversible capacity	LIB: 1259 mAh/g at 0.2 A/g	[61]
Si/C-CNFs-20	Carbon-coated nanofibers via electrospinning + pyrolysis	Buffers Si volume; good rate capability	Moderate capacity vs. others	LIB: 1215.2 mAh/g after 50 cycles at 5 A/g	[73]
SiC-Graphite-180	Graphite + pitch pyrolysis	Enhanced conductivity and structure	Irregular morphology	LIB: 602.4 mAh/g; 93.4% retention after 50 cycles	[76]
Si/PC-30	N-doped porous C via PAN + CaCO_3_ template	Porous shell, high conductivity	Complex synthesis	LIB: 830 mAh/g after 200 cycles	[81]
Si-Cu_3_Si-CNT/G-C	Cu_3_Si + CNT + graphite + C-coating	Excellent rate and cycle performance	Complex architecture	LIB: 1237 mAh/g; ~1000 mAh/g at 1C	[82]
Si@C@RGO	Dual carbon coating (rGO + C layer)	High capacity; reduced Si expansion	Synthesis complexity	LIB: 2124 mAh/g; 94.9% retention after 100 cycles at 200 mA/g	[91]
Si/graphene (1:4)	Graphene coating via spray drying + annealing	Improved rate capability	Less effective at high graphene %	LIB: 1298.1 mAh/g; better rate-capability at 1000 mA/g	[93]
Si/MWNTs	CVD of Si on MWNTs	Enhanced contact; high conductivity	Requires metal catalyst	LIB: 2049 mAh/g with only 19.7% capacity loss	[99]
Si@C-2 (CVD)	Uniform C-layer via rotational CVD	Stable at high rates; uniform coating	Longer CVD time	LIB: 1600 mAh/g (70 cycles); 750 mAh/g at 5 A/g	[104]
NG@SiC	N-doped graphene–SiC heterostructure	High rate capability and durability	Graphene prep needed	LIB: 1197.5 mAh/g (200 cycles); 447.8 mAh/g after 1000 cycles at 10 A/g	[151]
B-ASiCNR (DFT)	B-doping of armchair SiC ribbon	High Li affinity; strong bonding	Theoretical model only	LIB: 836 mAh/g (theoretical)	[115]
pSi/SiC spheres	Porous Si + SiC via magnesiothermic reduction	Excellent long-term durability; low resistance	Multistep synthesis	LIB: 1022 mAh/g (400 cycles at 2 A/g); 420 mAh/g after 2000 cycles at 5 A/g	[137]

## Data Availability

All the data are presented in this manuscript.
